# State of the Art and Challenges in Complete Benzene Oxidation: A Review

**DOI:** 10.3390/molecules29225484

**Published:** 2024-11-20

**Authors:** Tatyana Tabakova

**Affiliations:** Institute of Catalysis, Bulgarian Academy of Sciences, 1113 Sofia, Bulgaria; tabakova@ic.bas.bg

**Keywords:** air pollution, VOCs, benzene, catalytic oxidation, catalyst preparation, reaction kinetics and mechanism, catalyst deactivation

## Abstract

Increased levels and detrimental effects of volatile organic compounds (VOCs) on air quality and human health have become an important issue in the environmental field. Benzene is classified as one of the most hazardous air pollutants among non-halogenated aromatic hydrocarbons with toxic, carcinogenic, and mutagenic effects. Various technologies have been applied to decrease harmful emissions from various sources such as petrochemistry, steel manufacturing, organic chemical, paint, adhesive, and pharmaceutical production, vehicle exhausts, etc. Catalytic oxidation to CO_2_ and water is an attractive approach to VOC removal due to high efficiency, low energy consumption, and the absence of secondary pollution. However, catalytic oxidation of the benzene molecule is a great challenge because of the extraordinary stability of its six-membered ring structure. Developing highly efficient catalysts is of primary importance for effective elimination of benzene at low temperatures. This review aims to summarize and discuss some recent advances in catalyst composition and preparation strategies. Advantages and disadvantages of using noble metal-based catalysts and transition metal oxide-based catalysts are addressed. Effects of some crucial factors such as catalyst support nature, metal particle size, electronic state of active metal, redox properties, reactivity of lattice oxygen and surface adsorbed oxygen on benzene removal are explored. Thorough elucidation of reaction mechanisms in benzene oxidation is a prerequisite to develop efficient catalysts. Benzene oxidation mechanisms are analyzed based on in situ catalyst characterization, reaction kinetics, and theoretical simulation calculations. Considering the role of oxygen vacancies in improving catalytic performance, attention is given to oxygen defect engineering. Catalyst deactivation due to coexistence of water vapor and other pollutants, e.g., sulfur compounds, is discussed. Future research directions for rational design of catalysts for complete benzene oxidation are provided.

## 1. Introduction

**Figure 1:**
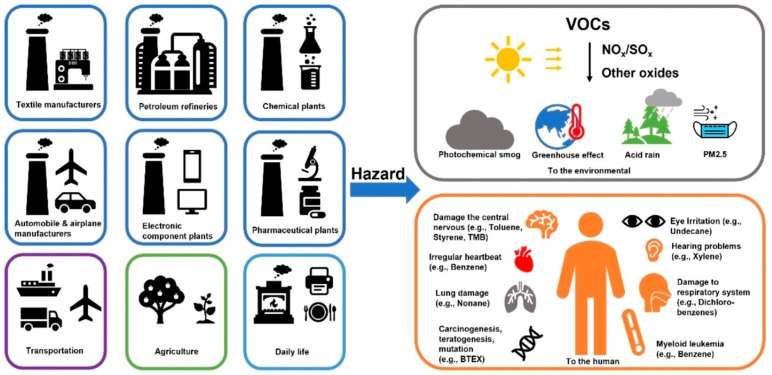
Main sources of VOC emissions and associated hazards to humans and the environment. Reproduced from [10]. This is an open access article distributed under the Creative Commons Attribution License.

During the last century, human activities contributed significantly to improving quality of life. At the same time, industrialization and urbanization resulted in increasing emissions of volatile organic compounds (VOCs), which are an important class of atmospheric pollutants of urban and industrial areas [1]. VOCs are a class of organic compounds with low boiling points (<250 °C) at atmospheric pressure, which enable vaporization at ambient conditions [2]. The Environmental Protection Agency defines VOCs as “any compound of carbon, excluding carbon oxides, carbonic acid, metallic carbides, inorganic carbonates, bicarbonates, and ammonium carbonate, which participates in atmospheric photochemical reactions” [3]. Many VOCs are toxic, carcinogenic, and/or mutagenic, thus posing severe health risks [4]. Exposure to high concentrations of VOCs can severely affect the human respiratory and immune systems [5,6]. Formation of secondary organic aerosols by atmospheric oxidation and condensation of VOCs also contributes to air pollution and affects the survival of plants, animals, and humans. Some of the main sources of VOC emissions are automobile exhaust, industrial and energetic processes, solvent use, construction materials, residential activities, furniture and combustion by-products, etc. [7,8,9]. They are shown in Figure 1 together with their hazardous effects on humans and the environment [10]. Due to the wide diversity of sources, these hazardous pollutants affect both outdoor and indoor environments, causing detrimental effects on human health and eco-systems [11,12]. Air pollution is closely related to climate change and some effects of global warming are now occurring faster than scientists had predicted [13,14]. Many efforts should be focused on emission abatement in order to avoid some of the worst outcomes. During the 28th United Nations Climate Change Conference convened in Dubai from 30 November to 13 December 2023, more than 150 heads of states and governments recognized the importance of helping countries strengthen resilience to the effects of climate change [15]. 

### 1.1. Short Overview of VOC Abatement Technologies 

**Figure 2:**
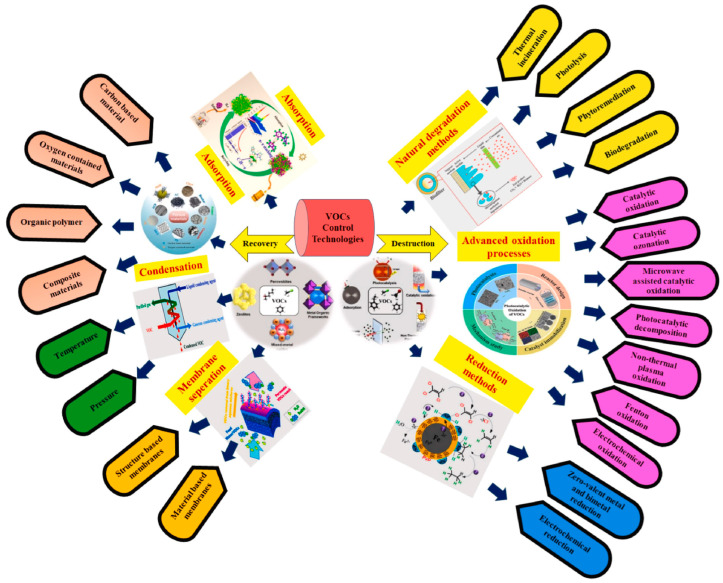
Schematic overview of VOC control strategies. Reproduced with permission from [23]. Copyright Elsevier B.V.

Various technologies have been developed and applied for VOC removal, aiming to comply with increasingly stringent emission regulations [16,17,18,19,20,21,22]. In a very recent review, Baskaran et al. provided a comprehensive understanding of the various methods used for controlling VOCs abatement, highlighting their respective pros and cons [23]. An overview of the VOC control strategies developed so far is shown in Figure 2. The first group is based on recovery and concentration and includes absorption, adsorption, membrane separation, and condensation. The degradation principle is the main feature of the second group, with technologies divided into natural degradation, advanced oxidation, and reduction methods. The most used are thermal incineration, catalytic oxidation, biological treatment, and photocatalysis. Among them, catalytic oxidation is an environmentally friendly and economically profitable approach, able to completely transform very dilute organic pollutants (less than 1%) to CO_2_ and water at relatively low temperatures. It is widely used owing to some important advantages such as high catalytic activity and treatment efficiency, low energy consumption, simple and easy operation, as well as no secondary pollutants [23,24,25,26]. 

Along with many papers, several comprehensive reviews cover recent developments and achievements by focusing on various aspects specific to catalytic VOC oxidation. These include design of catalytic systems with appropriate composition, effect of synthesis method, characterization by different techniques, relationship between structural, electronic and reductive properties of catalytic materials and their performance, stability and durability, resistance to deactivation, and reaction mechanism [4,7,8,9,10,25,26,27,28,29,30,31,32,33,34,35]. He et al. systematically summarized the effect of VOC types and sources on pollutant removal from industrial waste streams, providing an excellent base for rational catalyst design and future progress in the field [36]. 

### 1.2. Recent Progress in Development of VOC Oxidation Catalysts 

Considering the pivotal role of catalysts for achieving high efficiency of VOC abatement, many research efforts are devoted to catalyst design and composition optimization. Generally, two main types of catalysts are most intensively studied: supported noble metals, and transition metal oxides or mixed metal oxides [7,8,25,27,37,38]. Noble metal-based catalysts (Pd, Pt, Rh, Ir, Au, Ag) have emerged as a compelling solution, because in spite of their high cost they demonstrate excellent activity and selectivity at low temperatures, favorable resistance to deactivation, and easy regenerability [39,40,41,42,43]. In an excellent review, Liotta outlined the importance of noble metal nature and type of VOC on catalytic performance, focusing on variations in acidity, selectivity, and stability owing to preparation method, metal particle size, metal precursor salt, support porosity, presence of chlorine and water in waste gases, etc. [39]. Most commonly used catalysts for industrial VOC abatement are noble metals (Pt, Pd, and Rh) supported on γ-Al_2_O_3_ owing to its stability, high surface area, and excellent resistance to various poisons, or wash-coated on a monolith. Very recently, Chu et al. discussed in detail the reaction mechanisms and the effect of different factors such as preparation method, noble metal oxidation state and dispersion, and type of support on VOC abatement over Pt- and Pd-based catalysts, also analyzing briefly other noble metal (Au, Ag, and Ir) catalysts [43]. 

Among the noble metal-based catalysts, supported gold nanoparticles have attracted particular interest from the catalytic community over three decades owing to their remarkable ability to achieve complete conversion of various VOCs below 200 °C. Considerable efforts have been focused on exploration of the effect of synthesis approach on gold particle size and oxidation state, support effects, relationship between electronic and structural properties of the gold-based catalysts, and catalytic performance [44,45,46,47,48]. According to Scirè and Liotta [44], complete VOC oxidation over Au/metal oxide catalysts depended on many factors related to both the support and gold properties, between which a co-operative synergistic effect often existed. The nature of the support has been highlighted due to its crucial role for the size, shape, and amount of supported gold. Barakat et al. have discussed critically the application of non-porous, porous, and hierarchically porous metal oxides as supporting material, revealing how chemical composition, surface area, and physical architecture of the support affect interaction with gold particles and with pollutant molecules [45]. Reduction behavior of metal oxides has also been considered because of the opportunity for direct participation of reducible oxides in the reaction pathway. Carabineiro et al. have demonstrated that the reducibility of CuO, La_2_O_3_, MgO, NiO, and Fe_2_O_3_ is one of the major factors for enhanced oxidation of VOCs over supported gold catalysts [46]. The potential of ceria as a suitable support for gold catalysts has been reported owing to its excellent oxygen storage capacity, unique redox properties, and ability to provide high dispersion of supported metals. Very recently, Gaálová and Topka summarized published results for gold and ceria as catalysts for VOCs abatement, paying attention to optimal gold particle size and gold/ceria interface as main factors for enhanced activity and/or selectivity (see [47] and references therein).

However, some drawbacks of noble metals such as high cost, limited availability, and susceptibility to sintering have pushed research interest towards transition metal oxide-based catalysts. Metal (Co, Cu, Mn, Fe, Ni, Ce, Zr, etc.) oxides became attractive due to economic profitability as well as high performance that sometimes could be comparable to the noble metal-based catalysts if their design allows full access to the active sites [16,22,24,25,49,50,51,52,53,54,55,56,57,58,59,60,61]. Pan et al. have demonstrated that preparation of transition metal oxide-based catalysts with porous structures and tuned morphologies, such as nanorod, nanowire, nanotube, and nanoflower-like structures, contribute to exposure of more high-energy crystal planes with active sites, which improves reactants’ accessibility and enhances catalytic performance [50]. Recently, the role of special structure and properties of perovskite materials as one of the non-noble group of catalysts for VOC catalytic combustion has been revealed [28]. Synthesis methods and application of ceria with special morphologies such as hollow structures (spheres, fibers, and nanotubes), rod-like, cube-like, etc., as a support, promoter, and active component of CeO_2_-based catalysts for catalytic removal of air pollutants have also been reviewed [58,59]. Recent advances in layered double hydroxides for catalytic oxidation of VOCs owing to favorable properties such as tunable composition, uniform metal dispersion, large surface area, structural memory effect, low cost, thermal stability, and recyclability have been summarized by Yu et al. [62]. Unique physicochemical features of zeolites such as large specific surface area, strong acid sites, high adsorption capacity, high thermal/hydrothermal stability, and ability to adjust wettability and auxiliary mesopore formation contributes to successful application of zeolite-based materials in VOC abatement [63,64,65,66]. An attractive strategy to recover solid waste generated from coal combustion is the preparation of zeolites from coal fly ash. The potential of Cu- and Fe-modified coal fly ash zeolites for VOC oxidation has been properly demonstrated [67,68]. 

The advantage of combinations of noble metals and transition metal oxides due to enhanced electron transfer capability has also been explored [69,70,71,72,73]. A rational approach to reducing the consumption cost of precious metals is the selection of suitable carriers. Various mesoporous materials, such as silica-based, carbon-based, phosphate-based, and transition-metal-based with high specific surface area, unique porous structures, and uniform pore size distribution have been reported as superior supports for noble, transition, and rare earth metals or directly used as VOC oxidation catalysts [20,21]. Peculiar features of metal-organic frameworks (MOFs) such as high specific surface area, adjustable porosity, and unique topological structure make these materials very promising for elimination of gaseous pollutants. The latest progress and application of MOFs and their derivatives in catalytic oxidation of typical VOCs has been summarized [35,74,75]. Development of single-atom catalysts (SACs) has emerged as a successful concept for design of cost-effective catalysts [76]. During the last decade, SACs achieved significant progress in catalytic oxidation thanks to their unequaled advantages such as full exposure of active sites, tunable coordination, and strong metal-support interaction. SACs’ potential for effective elimination of various air pollutants was recently highlighted [77,78]. 

Bimetallic supported catalysts are also attractive due to higher atomic utilization by optimization of composition and morphology. Synergistic interaction between the two metals modifies surface electronic structures of the catalysts, thus beneficially affecting not only reactivity but also contributing to higher thermal stability and resistance to poisoning [79,80,81]. Hosseini et al. have found that catalytic oxidation of toluene, propene, and a gaseous mixture of both over Au-Pd supported on mesoporous TiO_2_ depends on the morphology of the core–shell structure, with the best performance shown by a combination of Au-rich core and Pd-rich shell [82]. Barakat et al. [83] have explored stability over 110 hours’ time-on-stream of a bimetallic Pd-Au entity supported on a hierarchical TiO_2_ doped by various elements. The niobium-doped sample exhibited cyclic toluene conversion related to the presence of hydroxyl radicals, coke molecules, and the redox behavior of Pd particles. Li et al. have prepared bimetallic core-shell structure nanoparticles, such as Ag@Pd and Au@Pd, thus optimizing the use of expensive Pd in the shell and reducing the cost, but also enhancing oxidation activity through electronic modification [84]. The combination of Pt and Pd on high surface area TiO_2_ synergistically affected the reaction rate and TOF (turnover frequency) in propane oxidation reaction, while stronger acidity of the bimetallic system was beneficial for catalyst water tolerance compared to monometallic counterparts [85]. Bimetallic Pt-Pd nanoparticles have been anchored on uniform mesoporous MnO_2_ nanospheres with large specific surface area and pore size, enabling an abundance of accessible active sites and enhanced diffusion properties [86]. The role of a secondary noble metal to tune the O_ads_/O_latt_ molar ratio, resulting in high toluene oxidation activity, has been revealed. 

This short overview emphasizes the important role of rational design in improving catalyst performance. The nature of different kinds of VOCs such as chemical structures, electronic environment, and physicochemical properties continues to be a great challenge in research efforts to find the best catalyst composition. Considering respectful achievements in the field of VOCs catalytic oxidation by many researchers, this review focuses on the recent development of highly efficient catalysts for effective elimination of benzene at low temperatures. Within the VOCs, benzene, toluene, ethylbenzene, and xylene belong to a group of compounds known as BTEX and are regarded as the most common aromatic VOCs with significant contribution to industrial emissions. Many studies select benzene as a target molecule for testing catalyst activity in aromatic hydrocarbons oxidation because of the extraordinary stability of its six-membered ring structure. Undisputed evidence of ever-growing interest is the increasing trend of published papers with keywords of “VOCs removal, VOCs catalytic oxidation, complete benzene oxidation” (based on Scopus and WoS database). Advances in catalyst composition and preparation strategy over the last decade are summarized and discussed. Advantages and disadvantages of using noble metal-based catalysts and transition metal oxide-based catalysts for complete benzene oxidation are addressed. Effects of some crucial factors such as catalyst support nature, metal particle size, reaction conditions, and electronic state of the active metal on benzene removal are explored. Benzene oxidation mechanisms are analyzed based on in situ catalyst characterization, reaction kinetics, and theoretical simulation calculations. Some aspects of catalyst deactivation such as water, sulfur, chlorine poisoning, coking, and sintering are noted. Oxidation of benzene in mixture with other VOCs is also described.

## 2. Properties and Sources of Benzene

Benzene (C_6_H_6_), an aromatic hydrocarbon, is one of the most common VOCs causing indoor and outdoor air pollution. It is a colorless or light yellow transparent oily liquid able to volatilize readily at room temperature with a strong aromatic odor [87]. Its lifetime in air ranges from a few hours to some days and depends on the presence of other pollutants and ambient conditions [88]. Benzene is considered very hazardous to the environment and human health. Since 1979, the International Agency for Research on Cancer has classified benzene as carcinogenic to humans based on sufficient evidence that it causes leukemia, particularly increasing the risk of acute myeloid leukemia [89]. Benzene exposure detrimentally affects the neurological, respiratory, immunological, and reproductive systems [90]. Linet et al. have reported that more than two million workers worldwide are exposed to benzene each year [91]. The authors concluded that chronic benzene exposure was responsible for a substantial increase in the risk of myeloid and lymphoid neoplasms, lung cancer, and respiratory diseases. Lifetime cancer risk for benzene was evaluated in roadside and along the traveling routes within urban and suburban areas of the Bangkok Metropolitan Region [92]. The highest risk was found for pickup drivers and in urban areas rather than in the suburbs.

Historically, benzene has been a key chemical component in many industrial processes and residential activities. Petroleum refining processes such as crude distillation, cracking, catalytic reforming, coking, refinery gas processing, etc., emit waste streams with a wide variety of VOCs including benzene. Industries such as production of organic chemical raw materials, textile dyeing, and printing also contribute to increased levels of benzene in waste emissions. The use of large quantities of organic solvents in leather manufacturing processes discharges a significant amount of VOC pollutants, with benzene being one of the main components. The pharmaceutical industry, pesticide production, and surface coating process as a stage for production of a variety of equipment and tools such as automobiles, motorcycles, bicycles, ships, containers, furniture, household appliances, etc., also release large amounts of VOCs into the atmosphere, including mainly benzene. Benzene is one of the main pollutants emitted from the three discharge sections in electronic equipment production processes, namely semiconductor and integrated circuit manufacturing, and printed circuit board and electronic terminal product production [36]. 

## 3. Catalysts for Complete Benzene Oxidation

Noble metal and non-noble metal catalysts are widely studied for application in complete benzene oxidation, similarly to the above-mentioned two main groups of materials for catalytic removal of VOCs. However, most of these works have not been reviewed, since the scope of this paper is to present an overview of the state of the art and progress during the last decade.

### 3.1. Noble Metal-Based Catalysts 

Generally, the performance of supported noble metal (e.g., Pt, Pd, Rh, Ru, Au, and Ag) catalysts is governed by particle dispersion, chemical state, and location of active sites, which are influenced by various factors such as preparation method, precursor type, particle morphology, and nature of the carrier. Selection of supporting material is an important task since it can strongly affect nanoparticle size, loading, and dispersion. It has been proved that catalyst activity and stability highly depend on carrier physicochemical properties, such as surface area, acid/basic characteristics, reducibility, and ability to enhance surface oxygen mobility [39]. 

#### 3.1.1. Platinum-Based Catalysts

Pt-based catalysts are well established due to their favorable properties for oxygen activation. Activated oxygen species play a beneficial role in cleaving the stable delocalized π bonds and strong C–H bonds of benzene. Commercial γ-Al_2_O_3_ as either a monolith or pellets is widely used for catalyst support in industry owing to its low cost, high thermal stability, mechanical strength, and high specific surface area. Li et al. have studied the effect of different morphologies of platinum nanoparticles on benzene oxidation [93]. Dendritic Pt/Al_2_O_3_ catalysts were prepared by a solution-based approach, and abundance of active surface-adsorbed oxygen was confirmed by XPS and H_2_-TPR. The catalysts demonstrated a higher complete benzene oxidation activity as compared to a sample with spherical Pt nanoparticles. Jung et al. explored the influence of reducing agents on the morphology and structure of colloidal nano-sized platinum particles supported on γ-Al_2_O_3_ [94]. Regardless of whether hydrogen or NaBH_4_ was utilized, the use of colloidal particles resulted in a more effective catalyst for benzene oxidation compared with Pt/Al_2_O_3_ prepared by the conventional impregnation method. A good understanding of Pt particle size effect on the degree of complete benzene oxidation was illustrated by study of Pt/Al_2_O_3_ catalysts with particle size ranging from 1.2 to 2.2 nm prepared by the modified ethylene glycol reduction approach [95]. Superior performance of a sample with the smallest platinum particle size of 1.2 nm was explained by the ability to provide more active sites for benzene oxidation and facilitate formation of more adsorbed oxygen. 

Some authors have applied an attractive approach to diminish noble metal content and increase exposure of Pt surface atoms, and to prevent the sintering process by alumina modification with metal oxides. Commercial mesoporous γ-Al_2_O_3_ with different amounts of V_2_O_5_ (5, 7.5, 10, and 12.5 wt.% V) was promoted by Ce and Pt [96]. The addition of 10 wt.% Ce and 0.3 wt.% Pt significantly improved the catalytic combustion of benzene, owing to high dispersion of the active components on γ-Al_2_O_3_ as well as enhanced redox properties resulting from the metal–metal and metal–support interaction. The catalyst demonstrated a good potential for industrial application due to good durability and resistance to water and chlorine poisoning during a long-term test. Bimetallic PtM (M = W, Mo) catalysts with narrow particle size distribution and uniform morphologies were prepared via solvothermal synthesis and dispersed on alumina [97]. Highlighting the role of small MO_x_ structures in close contact with Pt in adsorbing and activating benzene emphasized their significant contribution. A stronger interaction between highly dispersed metallic Pt species and small MO_x_ ensembles explained good tolerance to chlorine poisoning. Li et al. achieved complete destruction of benzene at 150 °C over Pt/Al_2_O_3_ modified by 0.6 wt.% reduced graphene oxide (GO) [98]. X-ray photoelectron and Raman spectroscopic data provided evidence of electron transfer between reduced GO and Pt, highlighting the role of GO in oxygen activation and high catalytic activity, respectively.

CeO_2_ has been investigated as a support for Pt-based catalysts. Mao et al. prepared 1 wt.% Pt nanoparticles on mesoporous CeO_2_ and ceria nanocubes by means of impregnation followed by reduction with NaBH_4_ [99]. Theoretical and experimental study revealed that the partial confinement of Pt nanoparticles in the ceria mesopores related to significantly enhanced activity of the surface lattice oxygen at the Pt particle interface perimeter. In contrast to the common opinion of the beneficial role of higher Pt dispersion for catalytic activity, Liu et al. reported that ceria-loaded Pt nanoparticles exhibited higher benzene oxidation activity and lower activation energy than single-atom catalysts [100]. The claim for better suitability of supported Pt nanoparticle catalysts was based on experiments and density functional theory calculations indicating that the metallic state of Pt and its electron-rich frontier orbitals contributed to activation of benzene and O_2_, respectively. Ni et al. reported similar findings by providing comparative insights into the factors governing size effect in Pt/Fe_2_O_3_ catalysis [101]. They found that platinum’s metallic state and resulting electron density governed excellent benzene and oxygen adsorption capacities, rather than dispersion. However, Yang et al. demonstrated SACs’ potential for complete benzene oxidation by study of 3D mesoporous Fe_2_O_3_-supported single-atom Pt with 0.08, 0.15, and 0.25 wt.% Pt [102]. The superior oxygen activation ability of 0.25Pt/Fe_2_O_3_ due to strong interaction between single platinum atoms and meso-Fe_2_O_3_ and promoted by Pt adsorption of benzene contributed to a seven-fold higher rate than that calculated for other catalysts of the same platinum nanoparticle loading. Most probably, these discrepancies originate from differences in the preparation of Pt-based catalysts. A possible solution could be detailed elucidation of the relationship between structure–reactivity of diverse Pt dispersion levels, electronic configurations, and catalytic activity of well-defined Pt entities located on regularly faceted substrates.

Titanium dioxide was also shown as an efficient and effective carrier owing to its low cost, non-toxic nature, high thermal stability, and ability of acidic sites to assist CO_2_ adsorption/desorption. Kim et al. revealed the role of metallic Pt nanoparticles and Ti^3+^ species on TiO_2_ generated by hydrogen-based reduction pretreatment in enhanced benzene oxidation activity [103]. In a very recent study, Tian et al. prepared well-defined Pt nanoparticles with diameters ranging from 2.2 to 6.6 nm [104]. Fine-tuning the geometry of Pt nanoparticles and correlation between TOF and specific surface sites pinpointed the perimeters of Pt atoms as the prime active sites for benzene combustion. 

**Figure 3:**
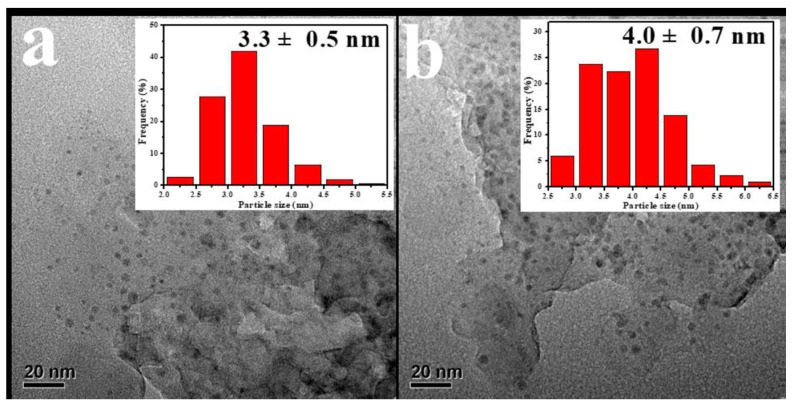
TEM images and the corresponding histograms of Pt NPs distribution (insets) of (**a**) bio-Pt/eggshell and (**b**) chem-Pt/eggshell. Reproduced with permission from [106]. Copyright (2020) American Chemical Society.

Based on the strategy of green and sustainable chemistry, diatomite-supported Pt catalysts were prepared by bioreduction method using *Cinnamomum camphora* leaf extract as reducing agent [105]. A catalyst composed of 0.3% Pt/diatomite exhibited complete benzene oxidation at 190 °C with no significant loss of activity during 60 h on-stream reaction time. Guo et al. [106] reported another example of fabricating supported Pt catalysts on environmentally friendly and cost-effective waste eggshell. The authors deposited highly divided and stable Pt nanoparticles through a plant-mediated biosynthesis method, in which *Cacumen platycladi* leaf extract was used for H_2_PtCl_6_· reduction. The eggshell-based bio-Pt catalyst significantly outperformed the chem-Pt/eggshell sample of the same Pt loading of 0.18% prepared by the excessive volume impregnation method. The advantage of employing plant-mediated biosynthesis was also demonstrated by comparing TEM images and matching histograms of platinum nanoparticles in Figure 3, where a much smaller average size was calculated in bio-Pt/eggshell (3.3 nm) than that of chem-Pt/eggshell (4.0 nm). Soybean straw as a renewable and abundant biomaterial was used as a carrier of 0.5 wt.% Pt nanoparticles [107]. The catalyst exhibited 90% benzene conversion at about 179 °C under a space velocity of 120,000 mL·g^−1^·h^−1^ and excellent catalytic stability. Application of coal fly ash zeolites as a good, cheaper, and environmentally friendly alternative to traditional zeolites synthesized from pure chemicals, produced an active platinum catalyst for complete benzene oxidation [108]. Simultaneous presence of redox pairs such as Fe^2+^/Fe^3+^, Pt^+^/Fe^3+^, and Pt^+^/Pt^0^ proved by XPS analysis contributed to easier oxygen release, thus improving complete benzene oxidation.

Optimization of Pt utilization through alloying with Co [109] or Fe [110] has been studied as an approach to overcome concerns with high cost and limited availability of noble metals. Platinum catalysts with various supports such as TS-1 [111], ZSM-5 [112,113], SBA-15 [114], Ti_3_C_2_ MXene [115], cryptomelane-type octahedral molecular sieve [116], mesoporous Co_3_O_4_ [117], La_2_O_3_ modified silica-pillared clays [118], antimony-doped tin oxide [109], and rare earths-doped MCM-41 [119] were also investigated. All these studies consider impact of preparation method, catalyst composition, experimental conditions on morphology and platinum particle size, Pt oxidation state and dispersion, abundance of active oxygen species, and redox properties. Table 1 gives comparative data about Pt-based catalysts used for complete benzene oxidation during the last ten years. However, an assessment of the best composition could not be done because of the diversity of experimental reaction conditions. 

#### 3.1.2. Palladium-Based Catalysts

Recently, Song et al. summarized advances in catalytic oxidation of VOCs over Pd-based catalysts [41]. Recent progress and developments in complete benzene oxidation were also described in this review. The relationship between catalytic performance of Pd-based catalysts and their composition, preparation conditions, Pd particle size and chemical state, and nature of supporting material were addressed [120,121,122,123,124,125].

Palladium oxidation state is often discussed because of controversial opinions about its role in benzene oxidation. Some authors claim that formation of metallic palladium particles provides more active sites for benzene oxidation relative to PdO [126]. Zhao et al. have prepared 3D-ordered mesoporous Co_3_O_4_-supported Pd nanoparticles by the one-step modified KIT-6-templating strategy. A 0.85-wt.% Pd/meso-Co_3_O_4_ catalyst was subjected to in situ reduction in a hydrogen flow at 200 and 350 °C. A superior activity for benzene combustion (*T*_90_ = 189 °C) of the sample reduced at 200 °C was attributed to the presence of metallic Pd particles, sufficient oxygen activation ability, and high surface area [127]. However, there are other viewpoints on the effect of palladium chemical state. Some authors ascribe better performance to catalysts with a mixture of PdO and Pd^0^ than to matching entities with only metallic Pd. Padilla et al. have reported that adding ceria to Pd/γ-Al_2_O_3_ enabled coexistence between Pd^0^ and PdO species, which favored benzene oxidation [128]. According to He et al., the mixture of Pd^0^ and PdO acts as active sites for benzene oxidation over Pd/SBA-15 catalyst [129]. The facile redox cycle of an active Pd^2+^/Pd^0^ couple in ultrathin Pd-W bimetallic nanosheets loaded on commercial TiO_2_ as well as atom-dispersed WO_x_ islands contributed to better catalytic activity and excellent water resistance in complete benzene oxidation in comparison with a supported Pd counterpart [130]. Study of the effect of pretreatment in different atmospheres (H_2_, N_2_, He, or air) on benzene degradation over Pd/γ-Al_2_O_3_ catalyst confirmed the importance of palladium’s oxidation state on catalytic activity [131]. Formation of metallic Pd species after pretreatment with hydrogen enhanced catalytic performance as compared to pre-oxidized catalyst with mainly PdO/Pd^2+^ species. An interesting finding was a favorable impact of inert atmosphere pretreatment on oxidation activity. Further characterization by XPS, Raman, and XRD techniques revealed that pretreatment in N_2_ or He caused modification of the crystal structure of the active species, with transformation of the PdO particles to amorphous state, thus beneficially affecting catalytic activity [132]. Modification of Pd/Al_2_O_3_ with Mo by impregnation effectively improved catalyst performance, which was attributed to coexistence between Pd^0^ and Pd^2+^ on the surface as well as to highly dispersed active Pd components and increased concentration of surface oxygen species [133].

Various Pd-containing compositions have been developed aiming at preparation of catalysts of high palladium dispersion. Given the advantageous features of perovskites, such as excellent thermal and structural stability, Yi et al. studied complete benzene oxidation over 0.5 wt.% Pd-CeMnO_3_ catalysts prepared by sol-gel and impregnation methods [134]. The authors explained the high efficiency of this formulation by formation of highly dispersed palladium particles on the perovskite surface and facilitated production of active oxygen resulted from interactions between Ce ions and Pd nanoparticles. Addition of 10 wt.% CeO_2_ to mesoporous silica-pillared clays with supported PdO_x_ was reported to produce an active catalyst for benzene oxidation due to increased oxygen vacancy concentration and improved Pd dispersion [135]. Higher Pd particle dispersion, stronger acidity of the ceramic fiber substrate subjected to leaching with sulfuric acid solution prior to Pd loading, and more active surface oxygen species initiated superior performance of 0.8 wt.% Pd catalyst in comparison with a glass-supported fiber sample [136]. 

The strategy for reduction of catalyst production costs stimulated development of bimetallic Pd-Cu nanoalloys with deposition on γ-Al_2_O_3_ and subsequent oxidation of the Cu component in alloy particles by thermal treatment [137]. Evaluation of catalyst performance indicated that both interaction between Pd and CuO and palladium dispersion in CuO played a decisive role in complete benzene oxidation. Hou et al. applied a successful approach to synthesize a bimetallic single-atom PdCo/Al_2_O_3_ catalyst with high dispersion of Pd and Co single atoms and double active site effect of Pd and CoO on the catalyst surface, resulting in an enhanced catalytic performance for benzene oxidation and superior SO_2_ resistance [138]. 

**Figure 4:**
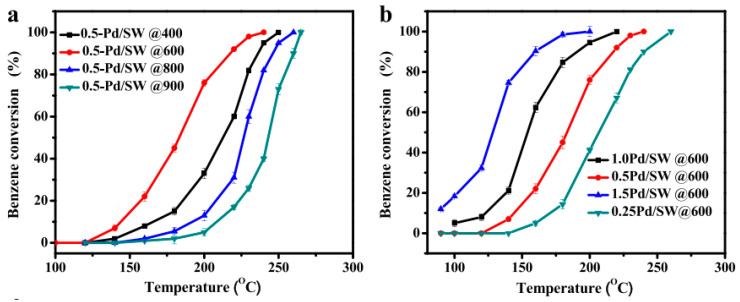
Complete benzene conversion over Pd/SW (shrimp waste) catalysts calcined at diverse temperatures (**a**); with diverse Pd loadings (**b**). Reproduced with permission from [124]. Copyright (2020) American Chemical Society.

Following the principles of green chemistry, Guo et al. applied biogenic synthesis with *Cacumen platycladi* leaf extract to produce Pd nanoparticles on 3DOM CeO_2_ [139]. Catalyst characterization and catalytic measurements revealed that a residual leaf extract protected Pd agglomeration and contributed to high dispersion of metallic Pd particles, abundance of adsorbed oxygen species, and strong interaction between palladium and ceria support. As a successful approach to waste utilization, Zuo reported the use of pearl shell powder from the pearl industry as a carrier of Pd particles [120]. The potential of shrimp waste (SW) as a cost-effective, green, and renewable carrier of supported palladium nanoparticles in the catalytic abatement of benzene was explored [124]. Benzene conversion over 0.5 wt.% Pd/SW catalysts calcined at diverse temperatures is shown in Figure 4a. The best performance of the sample calcined at 600 °C (*T*_90_ 235 °C) was ascribed to the highest O_2_ species content and relatively low reducibility compared to all tested catalysts. The catalytic activity was enhanced with increased Pd metal loading as a result of higher availability of Pd active sites and stronger metal-support interaction (Figure 4b). Waste red mud, commonly generated by alumina-related industries, was positively evaluated as a cost-effective support for Pd catalysts in complete benzene oxidation [140]. The effect of two different pretreatment procedures was studied, namely calcination and hydrochloric acid aqueous solution. It was found that Pd catalysts using HCl-treated material for support were more active owing to better mobility of surface lattice oxygen and presence of palladium oxide species. An overview of complete benzene oxidation over Pd-based catalysts is presented in Table 2. 

#### 3.1.3. Gold-Based Catalysts

Over the last decade, several works have reviewed the performance of gold-based catalysts in VOCs oxidation, focusing also on some achievements in complete benzene oxidation [44,45,47,48]. Different variables such as gold nanoparticle loading, size and oxidation state, type and physicochemical properties of support, in particular its reducibility, have been reported to affect the performance of gold-based catalysts for complete benzene oxidation. The role of gold for promoting lattice oxygen mobility to improve oxygen transfer to the vacancies and increase oxidation activity has been discussed. However, in many cases the relationship between some of the above-mentioned factors and the possibility of co-operative synergistic effect were considered to explain catalyst behavior.

Andreeva et al. have reported the earliest study of gold catalyst applicability for complete benzene oxidation [145]. Activity enhancement was ascribed to a synergetic effect between nanogold and partially reduced V_2_O_5_ supported on TiO_2_ and ZrO_2_ [146]. Some years later, the same group reported high and stable activity of ceria-supported gold catalysts promoted by vanadia [147] and molybdena [148], evidencing the suitability of nanosized gold for benzene removal and pointing to the importance of the support’s nature and metal-support interaction. Dai et al. have studied benzene oxidation over 3.2-nm gold particles supported on CeO_2_ bio-templated with bovine serum albumin (BSA) [149]. Characterization data indicated that ceria had a hierarchically porous structure, and higher pore size, pore volume, and specific surface area as compared with sample prepared without BSA. These properties favored generation of oxygen vacancies, and promoted synergetic effect of the support and highly dispersed gold particles. A benefit of strong gold-support interaction in high-temperature performance and durability of Au/Nb-CeO_2_ for benzene combustion reaction has been studied by Liu et al. [150]. Modification by highly dispersed and bulk NbO_x_ species caused lattice distortion and increased BET surface area, thus enhancing ceria redox capacity and facilitating abundant Au nanoparticles on the surface. 

**Figure 5:**
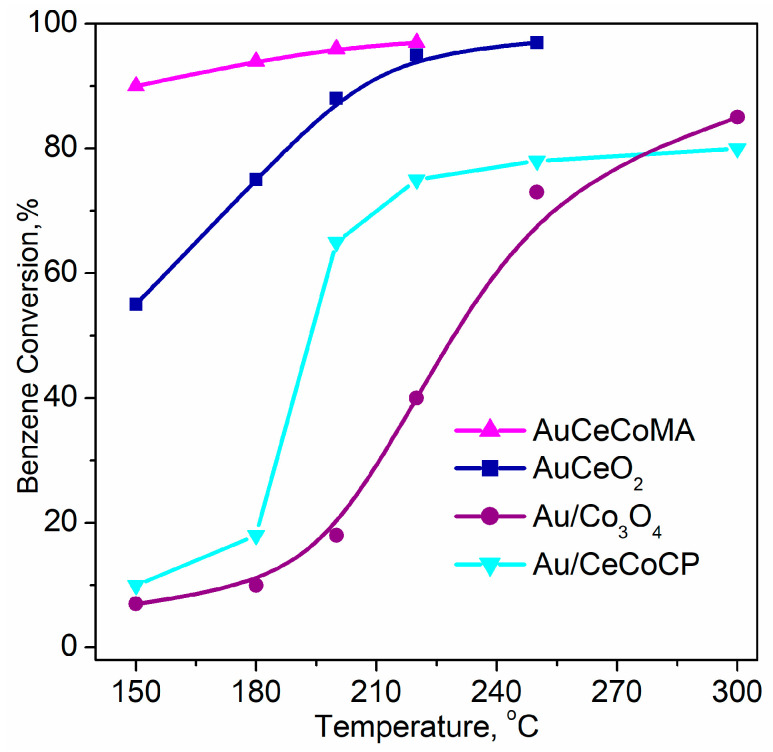
Complete benzene oxidation over gold catalysts: AuCeCoMA (▲), AuCeO_2_ (■), Au/Co_3_O_4_ (●), and AuCeCoCP (▼). Reproduced with permission from [151]. Copyright Elsevier B.V.

Ilieva et al. employed two methods, namely coprecipitation (CP) and mechanochemical activation (MA) for preparation of ceria doped with 10 wt.% Co_3_O_4_ aiming to improve benzene oxidation activity of an Au/CeO_2_ catalyst [151]. Mechanochemical activation produced a more active catalyst showing performance related to a double phase structure of the support, such as a separate Co_3_O_4_ phase and mostly surface-modified ceria (Figure 5). The authors highlighted the influence of highly dispersed gold and modified ceria in close vicinity on CoO_x_-phase reducibility, thus unraveling an important role of sample redox ability in the supply of active oxygen and oxidation activity, accordingly. 

**Figure 6:**
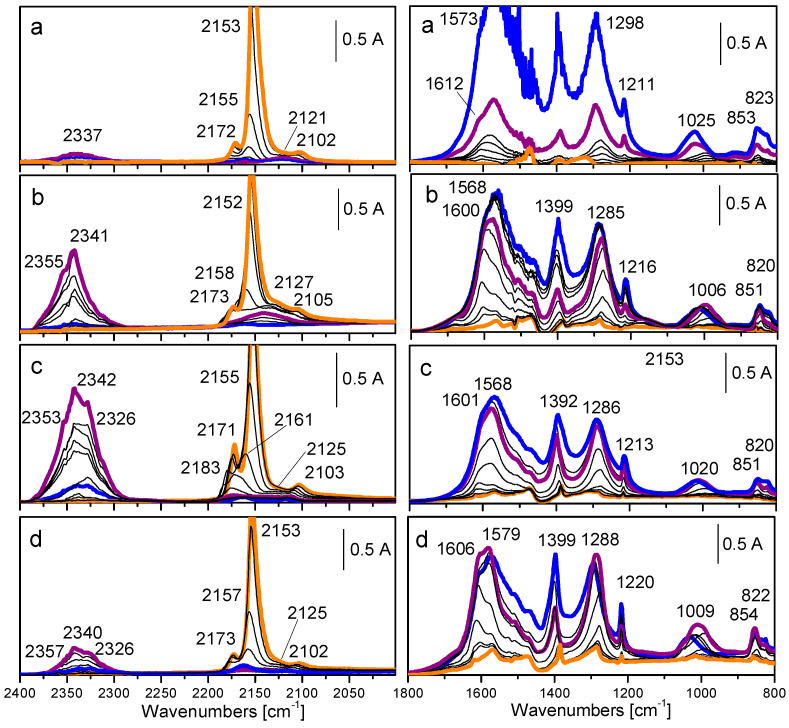
Evolution of FTIR difference spectra collected on Au/CeO_2_ (section **a**), Au/Ce5Co (section **b**), Au/Ce10Co (section **c**) and Au/Ce15Co (section **d**) immediately after the inlet of ^18^O_2_ at −180 °C on preadsorbed CO (orange lines) and at increasing contact times and temperature (black and purple lines) up to room temperature (blue lines) in the 2000–2400 cm^−1^ (**left**) and in the 1800–800 cm^−1^ (**right**) regions. Reproduced from [153] with permission from the Royal Society of Chemistry.

Further investigation of the effect of Co_3_O_4_ content in ceria carrier, namely 5, 10, and 15 wt.%, confirmed Au/CeO_2_–10Co_3_O_4_ superiority [152]. The highest reducibility and T_max_ shift to lower temperature in the TPR profile of this sample in combination with formation of gold particles of the smallest size determined the best benzene oxidation activity in comparison with ceria-supported gold catalysts doped with 5 and 15 wt.% Co_3_O_4_. Manzoli et al. reported very interesting spectroscopic results of the effect of support composition on active lattice oxygen supply [153]. Evolution of the bands in FTIR spectra during interaction between CO and ^18^O_2_ is shown in Figure 6. The intensity of the bands related to CO_2_ formed during CO oxidation follows the same trend as for the catalytic activity in benzene oxidation: AuCe10Co > AuCe5Co > AuCe15Co > AuCe. In more detail, three isotopomers of the CO_2_ molecule were registered: a growing band at 2326 cm⁻^1^, ascribed to the C^16^O^18^O solid-like phase, accompanied by bands at 2340–2342 and 2353–2357 cm⁻^1^ attributed to C^16^O_2_ and C^18^O_2_ solid-like phases, respectively. Analysis of the spectra disclosed that a fast exchange between oxygen of ceria and gas phase ^18^O_2_ molecules occurred even at a low temperature. However, the high intensity of the band ascribed to C^16^O_2_ provided evidence for increased oxygen mobility owing to enhanced ability of Co-promoted ceria to supply active lattice oxygen.

Additionally, fast Fourier transform analysis of HRTEM images of Au/Ce10Co and un-doped Au/CeO_2_ was performed [154]. In total, 1402 spacings were counted for Au/CeO_2_, with 89.23% of the spacings related to cubic CeO_2_ and 10.77% due to the defective and more reduced ceria phases Ce_6_O_11_ and Ce_2_O_3_. In the case of AuCe10Co, the number of spacings was 1212, among which 897 related to ceria phases and 315 were associated with cobalt oxide. Comparison revealed decreased amount of the cubic CeO_2_ spacings (62.65%) and increased amount related to defective ceria phases up to 37.35%, being almost four times higher than that observed on Au/CeO_2_. These results confirm the effect of the cobalt oxide phase on defectivity of ceria exposed sites, thus affecting reducibility and catalytic activity. Similar findings for correlations between low-temperature reducibility, high oxygen vacancy concentration, and activity for benzene oxidation of Au/meso-Co_3_O_4_ were reported [155]. Given the evolution of surface oxygen species depending on facet structure and composition, Jiang et al. pointed out the importance of surface concentration of oxygen ad-species for determining benzene oxidation performance of gold supported on Co_3_O_4_ hexagonal plates, with the most active (112) exposed facet tuned by Fe or Mn doping [156]. 

Manganese oxide suitability as a carrier of gold nanoparticles was also studied. Ye et al. have fabricated 3D-ordered mesoporous β-MnO_2_ and used NaOH, Na_2_CO_3_, or urea for deposition-precipitation of gold [157]. While gold loading affected reducibility via strong interaction between gold nanoparticles and β-MnO_2_ nanodomains, it was observed that synthesis conditions, in particular nature of the precipitating agent, controlled actual gold content. Remarkable enhancement in benzene oxidation activity of Au/Mn_3_O_4_ catalysts was also related to synergistic effect of different Mn_3_O_4_ morphologies and gold nanoparticles [158]. Significance of the Au-SnO_2_ interface for benzene combustion was studied by deposition of gold of narrow particle size distribution on SnO_2_ supports with morphologically uniform rhombic dodecahedra, elongated octahedral, and SnO_2_ octahedra [159]. A beneficial role of Au-SnO_2_ (111) interfacial structure in complete benzene oxidation was explored owing to the effect of gold-support interaction on surface/bulk lattice oxygen, Sn^4+^ valence state, and increased concentration of adsorbed oxygen species.

Very recently, the role of gold in complete oxidation of benzene over alumina-supported CuO-CeO_2_ mixed oxides of variable Cu/Ce ratio was reported [160]. Catalytic tests showed that a Ce-rich configuration outperformed the catalytic behavior of the single-component counterparts in the whole temperature interval. Incorporation of Cu ions into the ceria lattice was verified by XRD evidencing defect formation, particularly Ce^3+^ ions and oxygen vacancies. Promotion by gold and highly dispersed CuO_x_ provided additional benefits to the benzene conversion through enhanced oxygen mobility in ceria surface layers.

Bearing in mind the influence of specific interaction between gold and support on gold-based catalysts activity and stability, Wang et al. used hydroxyapatite (HAP) as a green and abundant material enabling stabilization of gold nanoparticles against sintering [161]. The authors attributed the origin of such a stabilization to interactions of the gold ions with phosphate and hydroxyl groups at the HAP’s surface. Functionalizing HAP surface with CeO_2_ and good contact between gold particles and both phases caused improved sintering resistance of nano-gold. Composition, reaction conditions, and complete benzene oxidation activities of some Au-based catalysts are listed in Table 3.

Preparation of bimetallic Au-containing catalysts is another approach to boost benzene oxidation and overcome the problem of gold nanoparticles’ aggregation at higher temperatures. Combination of favorable properties of gold and palladium was studied through preparation of Au-Pd catalysts on different supports. The best performance and most beneficial impact of Pd deposition on already deposited gold was observed with Pd-Au sample supported on CeO_2_ doped with Fe using the impregnation method [162]. Study of Pd, Au, and bimetallic Pd–Au supported catalysts on 1 wt.% Y_2_O_3_-doped ceria demonstrated that both noble metals synergistically enhanced support oxygen mobility, reflected in improved benzene conversion over the bimetallic catalysts with respect to the monometallic samples [163]. Modification by yttrium contributed to a further increase of support reducibility. XPS analysis of the oxidation state of the noble metals after catalytic tests confirmed reaction redox character, suggesting interaction of benzene with electron-depleted Pd^2+^ species and impact of lattice oxygen to palladium re-oxidation. The authors extended this study, aiming to develop catalytically effective and economically profitable materials. They prepared alumina-supported ceria and alumina-supported Y-doped ceria, both for use as support of mono- and bimetallic Pd-Au catalysts [164]. However, in contrast to previous finding of superior complete benzene oxidation activity of bimetallic Pd-Au catalysts, the best performance was demonstrated by a Pd-based sample on alumina-supported Y-doped ceria. It was suggested that alumina modification by 30 wt.% Y-doped ceria was not enough to provide an optimum number of active sites at the interface of gold and ceria for oxygen and C–H bond activation, while the catalytic performance of Pd-based catalysts was governed by metal-support interaction of Pd dispersed on Y-doped ceria and Pd located on Al_2_O_3_. Pd deposition over preliminary prepared Au/Y-CeO_2_-Al_2_O_3_ limited contact between Pd and ceria, because these sites were already occupied by gold. 

#### 3.1.4. Silver-Based Catalysts

Silver supported catalysts have also attracted research interest due to relatively low cost and high catalytic performance. Ma et al. prepared Co_3_O_4_-supported silver catalysts via a facile one-pot solvothermal method. The catalysts manifested a higher activity for benzene oxidation in comparison with Ag/Co_3_O_4_ synthesized by the impregnation method owing to abundant surface oxygen vacancy, high active oxygen species, and excellent low-temperature reducibility [165]. Higher contents of surface Co^3+^ and adsorbed oxygen species improved reducibility, and more active surface-lattice oxygen species reasonably enhanced complete benzene oxidation activity of silver loaded on Co_3_O_4_ by the reduction method [166]. Incorporation of Ag species strengthened interfacial electron transfer between Co and Ce, thus increasing surface Co^3+^ and oxygen vacancies [167]. Favorable properties of manganese oxides, such as excellent redox ability, tunable oxidation state (Mn^2+^, Mn^3+^, Mn^4+^, and Mn^7+^), diverse crystal phases, and high oxygen storage capacity made them an attractive support for silver particle deposition. Effect of Ag/MnOx preparation method, namely reduction, impregnation, or solution combustion, on benzene elimination was studied [168]. Reduction method caused a higher surface Mn^4+^/Mn^3+^ ratio, stronger reducibility, and more active surface oxygen species than other methods that resulted in better catalytic performance. Incorporation of well-dispersed Ag^+^ into cryptomelane-type manganese oxide preserved the cryptomelane structure but caused decreased crystalline size, increased surface area, and a higher number of Mn octahedral defects [169]. Very high benzene combustion was related to the formation of Ag-O-Mn entities and a large amount of active oxygen species. Chen et al. anchored isolated Ag atoms on the surface cavities of nanostructured manganese oxide [170], and observed enhanced catalytic performance in benzene oxidation owing to facilitated activation of gaseous oxygen by silver and an increased amount of active surface lattice oxygen. Co-deposition of Ag and Mn oxide on mesoporous ZrO_2_ nanofibers was developed as a catalytic material with better benzene oxidation performance at lower temperatures than that of Ag/ZrO_2_ and Mn/ZrO_2_, which was attributed to formation of active oxygen species and different manganese-oxidation states [171]. Bimetallic Ag-Ni catalysts were prepared by impregnation using boron-carbon-nitrogen (BCN) aerogel [172]. Large specific surface area and total pore volume of sample calcined at 400 °C favored high dispersion of Ag and Ni as well as adsorption and activation of reactants, while surface-adsorbed oxygen species and Ag/Ni-C/N bonds facilitated benzene oxidation. This sample attained 98% benzene removal within 6 h at room temperature; however, space velocity is not reported. 

A benefit of functionalized ZSM-5/SBA-15 support to produce coated silver nanoparticles located inside and outside the mesoporous system was reported by Nam et al. [173]. Support structural features provided increased active contact sites between reagents and silver nanoparticles. An efficient approach to mitigate environmental pollution was proposed by Liu et al. [174]. They modified LiCoO_2_ from spent Li-ion battery cathodes with AgNO_3_ and phosphotungstic acid (HPW) and observed increased number of surface oxygen vacancies and greater reactivity of surface oxygen species. Moreover, Ag addition improved benzene adsorption capacity, while both Ag and HPW contributed to strong binding strength of the benzene molecules with the catalyst surface, thus enhancing benzene oxidation activity.

**Figure 7:**
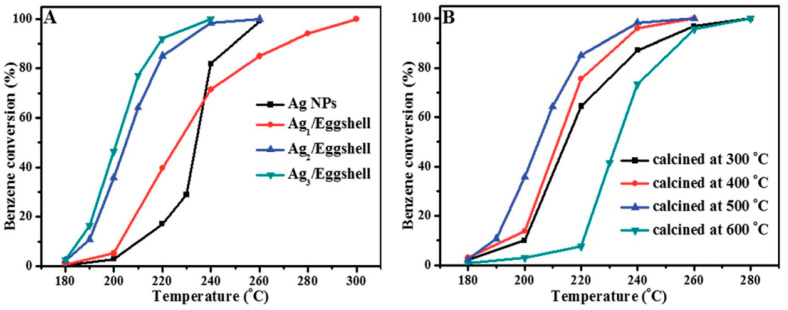
Temperature dependence of benzene conversion over (**A**) pure Ag NPs and Ag_x_/eggshell catalysts (x = 10.8, 19.9, and 34.3 wt.%) and (**B**) effect of calcination temperature of Ag_2_/eggshell at GHSV of 20,000 mL·g^–1^·h^–1^. Reproduced from [175] with permission from the Royal Society of Chemistry.

Guo et al. demonstrated a promising application of waste eggshells as a template and support of well-dispersed silver nanoparticles for benzene degradation [175]. A novel waste-derived Ag/eggshell catalyst exhibited excellent catalytic activity attributed to eggshell’s unique structure, good low-temperature reducibility, and high particle dispersion on eggshell material (Figure 7). Synergetic interaction between silver nanoparticles and eggshell was also highlighted. 

Table 4 presents a summary of the composition and catalytic performance of Ag-based catalysts reported in the reviewed literature. 

#### 3.1.5. Other Noble Metal-Based Catalysts

Only a few papers described development of Ru catalysts for complete benzene oxidation, probably because they used to show somewhat lower activity than Pt and Pd catalysts. An effective approach to increase the activity of a Ru catalyst by doping with a transition metal oxide has been reported by Liu et al. [176]. Samples of 1 wt.% Ru and 5 wt.% MO_x_ (M = Mn, Co, Ce, Cu, Fe) were supported on TiO_2_ through the impregnation method. Addition of Co_3_O_4_ led to preparation of the best-performing catalyst. Catalytic activity correlated well with enhanced reducibility of the cobalt oxide and XPS-derived highest O_ads_/O_latt_ molar ratio, implying a synergistic effect between Ru and Co. Complete benzene oxidation was examined by Sun et al. on Ru-doped CeO_2_ prepared via the one-step hydrothermal or impregnation method [177]. It was found that hydrothermal synthesis enabled Ru incorporation into the ceria lattice by replacing Ce atoms, resulting in a higher number of Ce–O–Ru bonds and surface oxygen vacancies in comparison with impregnation. In this case, enhanced redox processes and higher lattice oxygen mobility favored benzene oxidation at lower temperatures.

### 3.2. Non-Noble Metal-Based Catalysts 

The most commonly used non-noble metal-based catalysts include transition and rare earth metal oxides owing to their ability to form oxygen vacancies. Among them, Mn, Co, Cu and Ce oxide-based catalysts are mostly reported in the literature for benzene oxidation. 

#### 3.2.1. Manganese Oxide-Based Catalysts

Manganese oxides (MnOx) demonstrate a great potential for catalytic degradation of VOCs due to numerous advantages, such as multivalent oxidation states, different crystal phases and morphologies, good redox and catalytic properties, long-term durability, low cost, and abundance in nature. Very recently, published review papers have summarized the progress, opportunities, and challenges in catalytic oxidation of VOCs over Mn-based oxide catalysts [16,22,178]. These works have also covered some aspects specific to application of Mn-based catalysts in complete benzene oxidation. Xu et al. have classified manganese-based catalytic materials into four groups, namely single Mn oxide, supported Mn oxide, composite Mn oxide, and special crystalline Mn oxide [179].

In the case of single Mn oxide catalysts, the researchers have focused mainly on synthesis of different types of manganese oxides, materials of diverse structure, morphology, and exposed crystal planes. Zhang et al. utilized the hydrothermal method for preparation of 3D Mn_2_O_3_ with different morphologies such as cubes, fan-like structures, and spheres [180]. A sample of hierarchical cube morphology demonstrated the best catalytic performance with 90% benzene conversion at 252 °C, attributed to low crystallinity, high proportion of low-valence Mn^3+^ ions and O_latt_ species, and superior reducibility. Huang et al. prepared a series of Mn_2_O_3_ catalysts with 3D hierarchical cube-like morphology [181]. A sample subjected to hydrothermal treatment at 120 °C was the most active, with benzene conversion of 90% at 248 °C owing to highest amount of Mn^3+^ species and oxygen mobility. Improved low-temperature reducibility and abundant surface-adsorbed oxygen species were the main factors that determined the best catalytic activity of MnOx sample prepared via the citric acid solution combustion method at a citric acid/manganese nitrate ratio of 2:1 [182]. Li et al. [183] reported the same statements about the role of strong redox ability and abundant surface-active oxygen species in complete benzene oxidation. The authors studied porous λ-MnO_2_ with a spinel structure prepared from ZnMn_2_O_4_ via the acid etching route. Significantly higher benzene oxidation efficiency with *T*_90_ = 232 °C of octahedral layered birnessite-type manganese oxide with nanoflower morphology compared to nanowires and nanosheets was explained with highest lattice oxygen reactivity caused by a higher number of oxygen vacancies and Mn^3+^ [184]. Birnessite MnO_2_ with active oxygen vacancies and high performance was produced by acid treatment [185]. A promotional effect of acid surface sites on adsorption and activation of benzene and facilitated reactivity of lattice oxygen and surface-adsorbed oxygen favored the high activity of the acid-treated sample.

Wu et al. exploited the hydrothermal method for preparation of supported MnTiO_x_ catalysts with different Ti quantity [186]. The best oxidation performance was associated with 74.6% surface concentration of Mn^4+^ and availability of labile oxygen in surface layers. Among MO_x_/TiO_2_ (M = Mn, Ce, Co, Fe) samples, MnO_x_/TiO_2_ exhibited the highest catalytic efficiency, attributed to active oxygen species [187]. 

Many studies deal with composite Mn oxides because of their excellent catalytic activity related to synergistic effects, formation of more oxygen vacancies, and improved mobility of surface oxygen. Extensive research has been conducted for preparation of Mn-Cu mixed oxide catalysts with enhanced benzene combustion performance [188,189,190,191,192,193,194,195,196,197]. Generally, addition of copper to manganese oxide contributed to higher surface area [189,190,192,194], increased oxygen vacancy concentration [188,191,192,196,197], abundant surface-adsorbed oxygen species [189,191,194,196], and improved low temperature reducibility [189,190,191,197]. Because of high oxygen mobility, Co_3_O_4_ was also found to be an attractive additive. Adsorbed oxygen species and low-temperature reducibility of Mn–Co mixed oxide nanorods prepared by the sol-gel chelating method associated with strong synergistic effect between Mn and Co species in solid solution positively affected benzene conversion [198]. Creation of an MnO_2_-Co_3_O_4_ heterogeneous interface was achieved by decoration of three types of 1D MnO_2_, thus facilitating formation of surface active oxygen and improving low temperature reducibility [199]. Suitability of diatomaceous earth as a novel, cheap, and widely available support of MnO_2_-Co_3_O_4_ catalysts able to completely decompose benzene in the temperature range 225–250 °C was demonstrated [200]. 

Ceria’s excellent oxygen storage capacity and unique redox properties have motivated its addition to Mn-based catalysts for benzene degradation. Ce^3+^ incorporation into cryptomelane-type manganese oxide (OMS-2) has been studied by density functional theory (DFT) calculations and CO temperature-programmed reduction [201]. Location of Ce ions in tunnels produced a material exhibiting a higher catalytic activity than OMS-2 with Ce ions located in a framework because of decreased lattice oxygen activity. Mn–Ce composite oxides of different Mn/Ce molar ratios have been dispersed into the pores of silica spheres [202]. Synergistic effect between manganese and cerium oxide affected surface area, pore size and particle size distribution, reducibility, and benzene degradation activity. CeO_2_-modified manganese oxide supported on new kaolin-based NaY-type zeolite demonstrated a stable complete oxidation of benzene at 260 °C within 800 h, revealing a promising behavior for practical application [203]. Strong interaction between highly dispersed CeO_2_ and MnO_x_ on the zeolite surface resulted in higher amount of MnO_x_ at a higher oxidation state and improved reducibility. K-doped Mn–Ce solid solution catalysts exhibited 90% benzene conversion at approximately 194 °C that was comparable to that of noble metal catalysts [204]. Abundant oxygen vacancies provided by ceria and introduction of K^+^ caused weakening of the Mn–O bond, thus favoring enhanced activity. MnO_x_-CeO_2_ mixed oxides were homogenously dispersed into porous ceramic membranes by impregnation [205]. Supported MnO_x_-CeO_2_ of 3:1 atomic ratio showed *T*_90_ at 244 °C and high stability owing to the lower-temperature reducibility and the abundant active oxygen. Benzene decomposition was significantly improved by Ce incorporation into the lattice of birnessite-type MnO_2_ [206]. Ceria’s promotional role was related to formation of highly active lattice oxygen, oxygen vacancies, and surface-adsorbed oxygen. Electron transfer between MnO_x_ and CeO_2_, improved reducibility, and abundant surface lattice oxygen were considered to explain the high benzene oxidation activity of Ce-promoted Mn/Al oxide catalysts derived from hydrotalcites [207].

**Figure 8:**
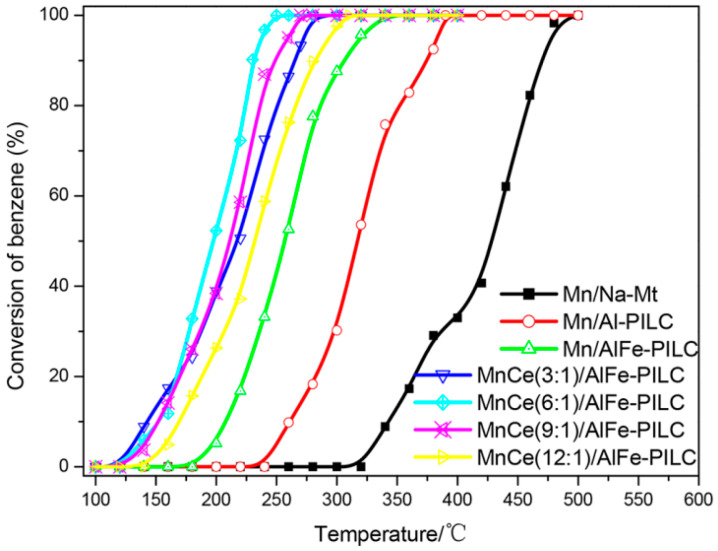
Benzene conversion over AlFe-pillared clay-supported catalysts with various Mn/Ce atomic ratios. Reproduced from [214]. This is an open access article published under an ACS AuthorChoice License.

Preparation of MnO_2_/NiO composites was reported as an effective approach to benzene low-temperature catalytic abatement [208,209,210]. Modification of MnO_2_ by Sm [211], Sn [212], and Sr [213] was another successful strategy for excellent benzene oxidation performance. Zuo et al. used AlFe-pillared clay as a perspective porous material for impregnation with Mn and Ce nitrate solution to produce a catalyst with 10 wt.% total amount of Mn or MnCe and Mn/Ce atomic ratios of 3:1, 6:1, 9:1, and 12:1 [214]. The authors discussed the effect of CeO_2_ addition on MnO_x_ dispersion and formation of an optimum number of oxygen vacancies for high and stable benzene oxidation at relatively low temperatures. Catalytic performance of catalysts with various compositions is shown in Figure 8. Mn-Ce(6:1)/AlFe-pillared clay demonstrated the highest activity and ability to completely degrade benzene at about 250 °C. 

#### 3.2.2. Cobalt Oxide-Based Catalysts

**Figure 9:**
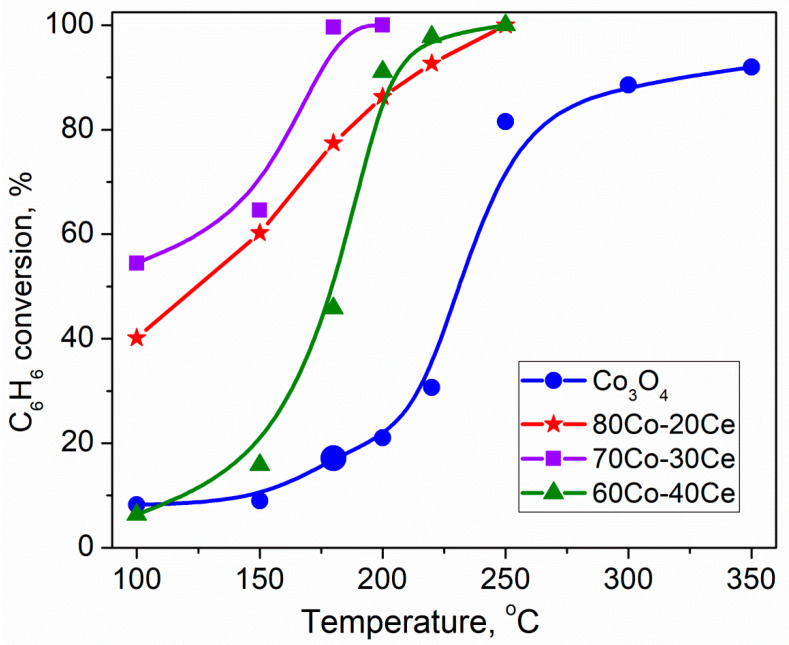
Comparison of benzene conversion over Co_3_O_4_ and Co-Ce mixed oxides. Reproduced from [220]. This is an open access article distributed under the Creative Commons Attribution License.

Cobalt oxides are also recognized as some of the most efficient catalytic materials used in complete oxidation of benzene. Advantageous features of Co_3_O_4_ are high reducibility, abundance of oxygen vacancies able to provide active surface oxygen species, and unique textural properties, as well as low cost, and chemical and thermodynamic stability. In a comprehensive review on the role of VOCs types and sources in air pollutants removal, He et al. briefly addressed preparation, composition, and catalytic performance of Co-based catalysts in benzene elimination [36]. Very recently, Chen et al. have summarized progress over the past decade in the design of cobalt-based catalysts for VOCs degradation, commenting also on the application of Co-based catalysts to catalyze benzene breakdown [215]. Some very recent achievements in benzene abatement over Co-based catalysts will be presented here for the sake of brevity. Zhang et al. reported preparation of a series of Co_3_O_4_ of different surface defective structures, focusing on the effect of the synthetic solvent used [216]. It was found that triethylene glycol strongly affected surface chemical structure and reducibility. The best catalytic activity, high stability, and good water tolerance were explained by the formation of more defective structure with abundant surface adsorbed oxygen and active lattice oxygen. Aiming to reduce the amount of cobalt oxide, which is unfortunately rather toxic, mixed metal oxides have also been investigated. Interfacial effects in CuO/Co_3_O_4_ with nanosheet-like heterostructures prepared by the wet chemical approach facilitated low-temperature reducibility and increased surface-adsorbed oxygen species [217]. CuO/Co_3_O_4_ catalysts can completely oxidize benzene under a high space velocity of 60,000 mL g^−1^ h^−1^ at 250 °C; that is 30 to 70 grad lower as compared to that of pure oxides, and demonstrated high stability over 10 continuous cycles and a 100-h time-on-stream test. According to DFT calculations, superior catalytic performance of CuO/Co_3_O_4_ was related to favorable benzene adsorption energy. Hydrotalcite-derived Co-Al mixed oxides exhibited different structures, morphologies, and redox properties due to strong interaction between cobalt and aluminum [218]. Calcination in N_2_ favored preparation of sample of much higher catalytic activity for benzene combustion and good long-term durability than air-calcined entity. Co_3_O_4_/α-Fe_2_O_3_ composites were prepared via coprecipitation and the effect of Co/Fe molar ratio and calcination temperature was examined [219]. Large specific surface area, high amount of surface oxygen species, and strong redox properties of a sample of 0.6 Co/Fe molar ratio calcined at 500 °C were beneficial for the best benzene oxidation. Ilieva et al. have prepared Co_3_O_4_-CeO_2_ composites (20, 30, and 40 wt.% of CeO_2_) by a simple, environmentally friendly, and less energy-demanding mechanochemical mixing [220]. All mixed oxides attained complete benzene oxidation at relatively low temperatures (200–250 °C) and demonstrated a significantly higher activity as compared to mono-component Co_3_O_4_ (Figure 9). Similar to the above-discussed effect of the Co_3_O_4_-CeO_2_ preparation method by co-precipitation or mechanical mixing on complete benzene oxidation over supported gold catalysts [151], mechanical treatment allowed obtaining surface-modified ceria and a separate Co_3_O_4_ phase with improved redox properties. Zhang et al. reported superior low temperature oxidation of benzene over Co_a_Mn_b_O_x_ nanosheets (a/b = 1:1~7:1) prepared by an oxalate co-precipitation method [221]. It was found that Co_2_Mn_1_O_x_ calcined at 300 °C displayed the best activity, achieving *T*_90_ at 191 °C under conditions of 20,000 mL·g^−1^·h^−1^ and 1500 ppm of benzene. As in many cases reported above, excellent oxidation activity was attributed to relatively high specific surface area, good low-temperature reducibility, abundant active oxygen species, and active components like Co^2+^ and Mn^4+^.

#### 3.2.3. Copper Oxide-Based Catalysts

Copper oxide also demonstrated good combustion activity. However, pure CuO is prone to deactivation because of its low thermal stability. SiO_2(1–x)_Cu_x_ catalysts with good reproducibility and low production cost were prepared by wet impregnation [222]. Samples were tested in the total oxidation of benzene within 50–350 °C, achieving over 85% at 150 °C. A promising performance for benzene removal was exhibited by compositions of mixed CuO with other metal oxides. Li et al. reported excellent catalytic activity towards complete oxidation of benzene of CuCo-based mixed metal oxides derived from Cu_x_Co_3–x_Al LDH precursors [223]. A sample of Cu_0.5_Co_2.5_Al composition reached 90% benzene conversion at 290 °C at a space velocity of 60,000 mL·g^−1^·h^−1^, in contrast to Cu3Al with only 8% conversion. Superior performance was attributed to high specific surface area, narrow pore size, low temperature reducibility, and rich oxygen vacancies and lattice oxygen derived from synergistic effect of CuO and Co_3_O_4_ spinel mixed oxides. These mixed metal oxides also demonstrated high water tolerance by maintaining 93.8% benzene conversion in the presence of 1.5 vol.% water as compared to 94.5% under dry conditions. Different methods such as combustion with malic acid [224], coprecipitation, urea-nitrate combustion, physical mixing [225], electrospinning, and surfactant-templating [226] have been employed for preparation of CuO–CeO_2_ mixed oxides. High dispersion of CuO, enhanced low temperature reducibility, and availability of weakly bonded oxygen species accounted for improved benzene removal. CuOx clusters and a Cu-[O_x_]-Ce structure were found as the main active sites for benzene combustion over Cu-Ce mixed oxides prepared through a sol-gel method [227]. 

#### 3.2.4. Ceria-Based Catalysts

**Figure 10:**
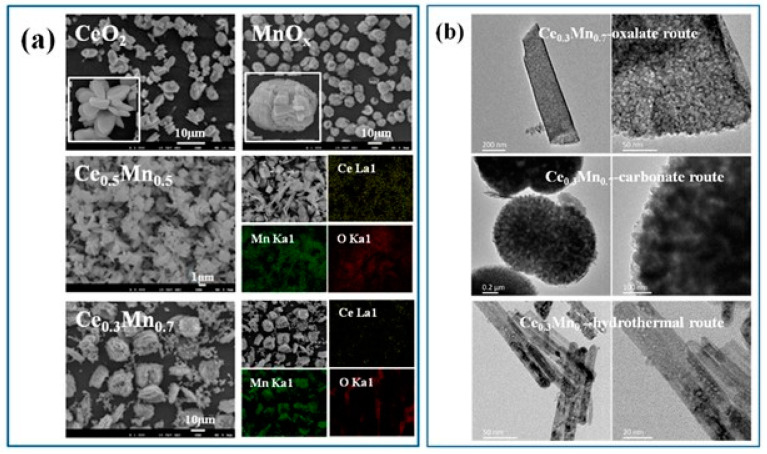
SEM images and EDX mapping of CeO_2_, MnO_x_, Ce_0.5_Mn_0.5_ and Ce_0.3_Mn_0.7_ (**a**); TEM images of Ce_0.3_Mn_0.7_ synthesized through different routes (**b**). Reproduced from [232]. This is an open access article distributed under the Creative Commons Attribution License.

Ceria was often used as a component and promoter for oxidation reactions due to a fast Ce^4+^ ↔ Ce^3+^ transfer, assuring oxygen storage and supply. Many works highlighted the role of surface layer oxygen and ceria vacancies in the catalytic activity, addressing the importance of synergistic effects between the components and oxygen vacancy defect engineering [59]. Complete benzene oxidation over various compositions comprising ceria and Mn, Co, and Cu oxides have already been discussed above. Mesoporous ceria prepared by thermal decomposition of Ce-MOF showed complete benzene oxidation at 260 °C [228]. Variation of Ce-MOF decomposition temperature affected ceria catalyst activity, with preliminary treatment at 400 °C being favorable for the largest specific surface area, pore volume, and highest activity. Huang et al. synthesized a cerium-based catalyst using cordierite honeycomb by a combustion method [229]. An MnCeOx/cordierite catalyst with Mn/Ce molar ratio of 1:1, Mn/(citric acid) molar ratio of 6, and subjected to thermal treatment for 7 h, demonstrated the best benzene conversion of 99.1% at 300 °C and space velocity of 20,000 h^−1^. Flame spray pyrolysis was applied to prepare Ce–Mn oxides, which were studied for catalytic oxidation of benzene [230]. Excellent catalytic activity of 12.5%-Ce–Mn oxide (*T*_95_ ~ 260 °C) was attributed to the small size of the catalyst particles and synergetic effect of Ce and Mn. Ke at al. have analyzed the role of defect sites on benzene removal over mesoporous ceria prepared by hard- and soft-template methods [231]. A highly defective internal structure and high specific surface area favored activity of ceria synthesized by the hard-template method below 300 °C. The role of preparation method was studied in several works. Ce_x_Mn_1−x_ composite oxides were fabricated by oxalate, carbonate and hydrothermal methods [232]. The best performance of Ce_0.3_Mn_0.7_ oxide with an atomic ratio of 3:7 (*T*_90_ = 190 °C) was related to sample microstructure due to the oxalate route of preparation. SEM analysis indicated formation of a large number of grains with layered structure (Figure 10a), while TEM images of Ce_0.3_Mn_0.7_ showed the presence of thin flakes (Figure 10b). Some mesoporous structures with mesopores size of ca. 2 nm were observed at the flake surface. Additionally, formation of a Ce-Mn solid solution facilitated creation of oxygen vacancies. CeO_2_/LaCoO_3_ was prepared by solid-state impregnation (SSI), electrospinning, and ball milling [233]. The highest concentration of surface-adsorbed oxygen species and oxygen mobility in the SSI-prepared sample was attributed to sufficient interaction between LaCoO_3_ perovskite and cubic ceria. Ceria impregnation with silica from rice husks as a low-cost and abundant agro-industrial waste was reported as a sustainable approach to preparation of benzene oxidation catalysts [234]. Ceria nanoparticle structure that is more open and has high dispersion produced the best result of ITQ-2 layered zeolite impregnated with 10% of Ce that attained 91% conversion at 350 °C and space velocity of 12,000 mL·g^−1^·h^−1^. Xia et al. studied the advantageous role of ceria in complete benzene oxidation of mesoporous Cr_2_O_3_ modified by oxides of Cu, Mn, and Co [235]. Interactions among Cr_2_O_3_, CeO_2_, and MnO_x_ produced highly active catalysts; a sample with 7.5 wt.% Mn was best-performing at *T*_90_ = 280 °C and space velocity of 20,000 mL·g^−1^·h^−1^. 

A summary of the composition and catalytic performance of some non-noble metal-based catalysts reported in the reviewed literature is presented in Table 5. 

#### 3.2.5. Mixed Metal Oxides Catalysts

There are some examples of mixed metal oxides of more complex composition adopted for complete benzene oxidation. In all cases, emphasis is given to designing catalysts of high specific surface area, improved low temperature reducibility and oxygen mobility, and availability of weakly bonded oxygen species. Deng et al. used co-precipitation for preparation of Co_x_Mn_1−x_CeO_δ_ mixed oxides [236]. Higher oxidation activity of Co_0.25_Mn_0.75_CeO_δ_ (*T*_90_ = 247 °C) was ascribed to the favorable effect of Co-doping on oxygen vacancy formation as well as to improved reducibility owing to strong interactions among all metal oxides. It was demonstrated that porous ceramic membranes (PCM) can provide more active sites for reactants by integration of Cu-doped Mn–Ce oxides by the sol–gel method [237]. Enhanced low-temperature reducibility, abundance of active adsorbed oxygen, and a higher number of surface oxygen vacancies determined the highest benzene conversion efficiency (*T*_90_ = 212 °C) of Cu_0.2_Mn_0.6_Ce_0.2_/PCM material. CuO, MnO_2_, and NiO were dispersed on the surface of Ce_0.75_Zr_0.25_O_2_ solid solution by incipient wetness impregnation [238]. Total loading of 10 wt.% of the three metal oxides produced a more active catalyst as compared to combinations between two metal oxides, with T_90_ at 250 °C and weight hourly space velocity of 72,000 mL·g^−1^·h^−1^. Ce incorporation into flame-made perovskite-type La_1−x_Ce_x_MnO_3_ (x = 0–10%) negatively affected perovskite’s specific surface area [239]. However, improved reducibility was observed owing to Ce^+4^-induced modification of the Mn^4+^/Mn^3+^ ratio and oxygen species concentration. Modification of perovskite-type oxide LaNiO_3_ with manganese by impregnation boosted benzene oxidation activity [240]. Increased Mn loading improved the redox properties and formation of Mn active sites on the surface.

## 4. Benzene Oxidation in Mixtures

An important question concerning industrial application of VOC oxidation is related to the presence of more than one pollutant in waste emissions depending on the process unit where they are emitted. All the above-reviewed works demonstrated the effectiveness of various catalytic materials for complete oxidation of benzene as a model of common aromatic VOCs. However, in many cases, VOC emissions comprise mixtures of air pollutants with quite different performance of each component. Some VOCs in industrial flue gas streams might occupy support active sites through chemisorption, thus decreasing the concentration of rate-determinant species. Recently, Rochard et al. summarized state-of-the-art effects that occurred during oxidation of VOC mixtures [2]. A detailed table displays effects such as inhibition and promotion, or lack of any effect. Some examples of benzene oxidation in mixtures over different catalytic materials are also discussed, considering both suppression of benzene oxidation or promotional effects of benzene on the oxidation of other VOCs. Zhang et al. studied simultaneous combustion of benzene and 1,2-dichloroethane (DCE) over bimetallic Pt-MO_x_ (M = W, Mo) catalysts [97]. Competitive adsorption between benzene and DCE molecules and an inhibitive effect of 50 ppm DCE on benzene oxidation at 180 °C were observed. However, a decrease in benzene conversion of 88 and 83% over PtW/Al_2_O_3_ and PtMo/Al_2_O_3_, respectively, was observed, as compared to 69% on Pt/Al_2_O_3_. No mixture effect was observed in the case of simultaneous oxidation of benzene and toluene over MOF-derived cobalt oxide modelled by computational fluid dynamics analysis and an artificial neural network [241]. Benzene and toluene conversion increased on raising the oxidation temperature in the range 150−300 °C. A slightly higher benzene conversion compared to that of toluene, i.e., 89.74 and 82.37%, respectively, was explained by differences in chemical structure and adsorption strength of both molecules. The opposite activity order with higher activity of toluene in binary mixture with benzene was found over 0.5 wt.% Ca/Mn_3_O_4_ catalyst [242]. Benzene was more active, showing *T*_90_ at 240 °C as compared to 260 °C for toluene conversion, while toluene was more readily removed in the binary mixture with *T*_90_ at 350 °C and 420 °C for benzene. An inhibitory effect between both compounds was suggested to depend on their adsorption properties [243]. 

Different mutual effects were observed on converting ethyl acetate, toluene, and benzene over Pd/ZSM-5 catalyst [244]. Benzene or toluene inhibited ethyl acetate oxidation, and both aromatic hydrocarbons inhibited each other. Ethyl acetate promoted toluene conversion, while an inhibitory effect on benzene oxidation was observed.

## 5. Benzene Oxidation Mechanism

**Figure 11:**
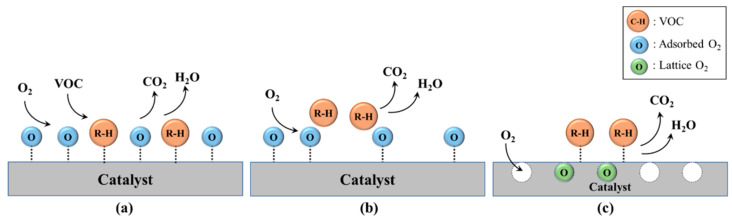
Graphic representation of the VOC catalytic oxidation reaction mechanism: (**a**) Langmuir–Hinshelwood, (**b**) Eley–Rideal, and (**c**) Mars–van Krevelen. Reproduced from [42]. This is an open access article distributed under the Creative Commons Attribution License.

Elucidation of reaction mechanism plays an important role in rational catalysts design. Generally, three different reaction mechanisms are proposed for VOC oxidation: Langmuir–Hinshelwood (L–H), Eley–Rideal (E–R), and Mars–van Krevelen (MvK) [30,39,42]. However, reaction mechanism depends on pollutant type, catalyst composition, and reaction conditions. The three mechanisms are schematically presented in Figure 11. According to the L–H model, the reaction occurs between adsorbed VOC molecules and adsorbed oxygen species (section a). Therefore, the reaction rate between these entities is the rate-controlling step. Usually, the noble metal acts as a bifunctional site for activation of oxygen and VOCs adsorption. The E–R model includes surface reaction between adsorbed reactants and gaseous molecules (section b). Firstly, oxygen molecules are adsorbed on the catalyst surface. Then, VOC molecules interact directly from the gas phase with the adsorbed oxygen species without adsorption. The MvK model assumes a two-step process for VOC oxidation. Firstly, adsorbed VOC molecules interact with oxygen from the catalyst, thus causing metal oxide reduction. The second step involves re-oxidation of the reduced metal oxide by the gas phase oxygen. The rate-determining step is the interaction between reactant molecules and oxidized sites at the catalyst surface.

**Figure 12:**
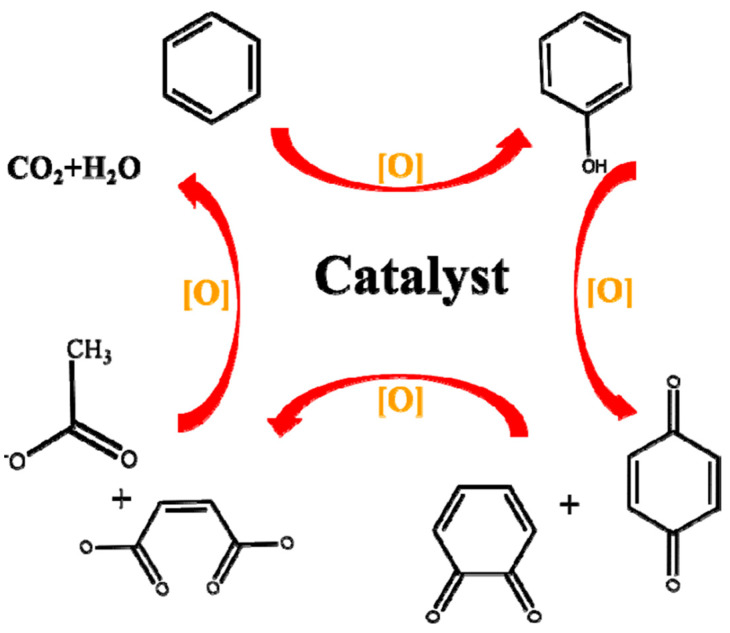
Reaction mechanism of benzene oxidation over the TiO_2_/PdW-S catalyst. Reprinted with permission from [130]. Copyright (2019) American Chemical Society.

According to Chen et al., benzene oxidation over Pt/Al_2_O_3_ catalysts follows the L–H mechanism [95]. Metallic Pt species are active sites for both benzene adsorption and activation and dissociative chemisorption of molecular oxygen. Interaction between adsorbed oxygen atoms and benzene leads to formation of carboxylate species that are decomposed into adsorbed CO and H_2_O. Adsorbed CO species eventually react with gas-phase O_2_ to produce CO_2_. In situ DRIFTS spectra were collected to determine intermediate species during benzene oxidation over TiO_2_/PdW-S [130]. It was suggested that, firstly, benzene oxidation proceeded by means of surface oxygen with formation of phenolate species, followed by their transformation of the latter into benzoquinone (e.g., o-benzoquinone and p-benzoquinone) species (Figure 12). Carboxylate (e.g., acetate and maleate) species were also produced by breaking the benzene ring. Finally, the carboxylate species underwent further oxidation to CO_2_ and H_2_O followed by catalyst recovery. Based on O 1s XP and in situ FTIR spectra, Guo et al. proposed a similar reaction mechanism for benzene oxidation over the Ag/eggshell catalyst [175].

Both MvK and L–H mechanisms were considered in the case of benzene oxidation over ACo_2_O_4_ (A=Cu, Ni, and Mn) spinel catalysts prepared by the co-nanocasting method using SBA-15 as a hard template [245]. Based on in situ DRIFTS study, participation of lattice oxygen in the formation of carboxylate intermediate species was proposed, while adsorbed oxygen species facilitated carboxylate species oxidation to final products. MvK mechanism was reported for benzene oxidation over alumina-supported Cu-Mn-Ce mixed oxide catalysts, where oxidation of benzene by surface lattice oxygen of the catalyst, and replenishment of the oxygen vacancy by oxygen to maintain the redox cycle, simultaneously occurred [246]. Catalyst composition of 30 wt.% CuO-MnO provided a high number of active sites and improved reducibility, while 4.4 wt.% CeO_2_ maintained high and stable dispersion of active metal, enhanced oxygen mobility, and oxygen vacancy formation. A study of benzene combustion over NiMnO_3_/CeO_2_/cordierite catalyst showed that the MvK mechanism explained well the kinetics of the oxidation process [247]. The authors concluded that the reaction occurred by interaction between the benzene molecules and active sites on the catalyst surface by a redox cycle of adsorption, de-oxidation, desorption, oxygen supply, and regeneration, the catalyst surface oxidation reaction being the rate-controlling step. Three kinds of oxygen species, namely, weakly bound oxygen species, surface-lattice oxygen species, and lattice oxygen species were found on MnO_2_ surfaces with different crystal phases [248]. A linear relationship was found between benzene oxidation rate and the amount of surface lattice oxygen, suggesting a decisive role of surface lattice oxygen species. Based on isotopic experiments, the MvK mechanism was proposed which includes benzene oxidation by surface lattice oxygen and subsequent filling of formed oxygen vacancies by gas-phase oxygen. 

The mechanism of benzene degradation at room temperature over AgNi/BCN was clarified by exploration of reactive oxygen species using EPR [172]. Spectral analysis detected the simultaneous presence of (•OH) and alkenyl carbon center radical (R•) resulting from activated oxygen and water in the air. The reactive oxygen species •OH is considered a key free radical for benzene ring opening at room temperature. Registration of (R•) revealed that ring opening led to formation of smaller olefin groups which further interacted with O_2_ and underwent isomerization to generate (•OH). Analysis of FTIR spectra before and after reaction provided additional evidence for participation of surface hydroxyls in the catalytic benzene oxidation that proceeded through benzene oxidation by (•OH) and formation of alcohols, carboxylic acids, and, finally, CO_2_ and H_2_O. Based on in situ FTIR experiments, the reaction mechanism of benzene oxidation over MnO_x_/TiO_2_ has been demonstrated [187]. Zeng et al. schematically presented the steps of benzene degradation. Firstly, benzene interacted with an active Mn center, producing phenolate species with two conjugated structures. Further, phenolate species were readily oxidized to form quinone species that underwent fast oxidation. Due to active oxygen species, ring opening gave rise to the formation of small molecule intermediates, such as maleate and acetate species. The last stage was oxidation of these species to final products of CO_2_, CO, and H_2_O. Active sites of the Co-based spinel catalyst were elucidated by several spectroscopic techniques: Raman spectroscopy, X-ray absorption fine structure spectroscopy, and in situ DRIFTS [249]. It was concluded that Co^3+^ species favor oxidative breakage of the benzene ring via interaction between σ and σ* C–H orbitals and Co^3+^ d-type orbitals, resulting in the formation of carboxylate intermediate species and their oxidation by oxygen to CO_2_ and H_2_O. Octahedrally coordinated Co^2+^ sites were found to be more easily oxidized by Co^3+^ species compared with tetrahedrally coordinated Co^2+^ sites. The highest surface amount of Co^3+^, Ce^3+^, and adsorbed oxygen species were evidenced by XPS and Raman analyses of the best-performing 70 wt.% Co_3_O_4_-30 wt.% CeO_2_ sample prepared by mechanochemical treatment [220]. The authors highlighted the importance of cooperation between Co_3_O_4_ and CeO_2_ for the benzene oxidation reaction. Synergistic interaction between cobalt oxide and ceria was described by the following mechanism: benzene causes partial Co^3+^ reduction to Co^2+^ or even Co^0^, followed by strongly enhanced re-oxidation by the neighboring ceria lattice, which is accompanied by creation of oxygen vacancies around the binary oxides interface, e.g., re-formation of sites for activated oxygen species generation.

Concerning benzene oxidation over zeolite-supported Pd catalysts, He at al. have proposed initial Pd^2+^O^2−^ reduction by benzene followed by Pd^0^ oxidation with oxygen from the stream and Pd^2+^O^2−^ recovery [250]. Such a redox process with gas-phase oxygen supply prevails for irreducible supports like alumina, whereas for reducible carriers like ceria, lattice oxygen from the ceria surface layers could participate in the redox transfer Pd^0^ ↔ PdO [164]. 

Shen et al. unraveled the benzene oxidation mechanism over Pd/Co_3_O_4_ catalysts with and without assistance of an electric field through analysis of DRIFT spectra [251]. In the case of conventional benzene oxidation, oxygen adsorption on the catalyst surface occurred first, followed by formation of active PdO_x_ species, which then interacted with gas-phase benzene through the E–R mechanism. However, reaction under an electric field enhanced oxygen mobility from catalyst bulk to catalyst surface. More active oxygen species appeared owing to Co_3_O_4_ reduction to CoO or Co^0^. Formation of PdO_x_ sites for benzene activation was also facilitated via the MvK mechanism. 

Theoretical modeling can provide useful information for catalyst design and prediction of catalyst performance. Calculations dealing with representative models are usually performed with the help of methods based on density functional theory (DFT). Results of DFT calculations found variations on increasing Pt–H and Ce–H bond length due to benzene adsorption on Pt single atom catalysts constructed by dual nanospace confinement of 3DOM CeO_2_ pore and Ce-MOFs nanocages [252]. Changes were also observed for C–C-H bond angle, indicating easier benzene adsorption on Pt_1_/CeO_2_@Ce-MOFs samples. DFT calculations indicated that C–C bond cleavage controlled benzene oxidation on a spinel-type CuMn_2_O_4_ catalyst [253]. Firstly, benzene dehydro-oxidation occurred to generate a phenoxy group (C_6_H_6_* → C_6_H_5_* → C_6_H_5_O*). Then, ring-opening and oxidation reactions of the phenoxy group proceeded by two reaction pathways, namely benzoquinone- and cyclopentadienyl-dominated. Finally, oxidation of C_5_H_4_O* and CO_2_ formation took place through a nine-step reaction pathway. The ring-opening reaction (C_5_H_4_O* → C_3_H_2_O*) was identified as the rate-determining step. Benzene oxidation on the surface of Pd particles was simulated by molecular dynamic simulation based on the ReaxFF force field [254]. It was concluded that benzene oxidation occurred firstly through dehydrogenation with C_6_HO as the main product. More probably, ring opening occurred at oxygen-containing site, followed by formation of smaller molecules. 

## 6. Effect of Water Vapor and Other Impurities on Benzene Removal Efficiency

In summarizing research efforts for rational preparation of highly efficient benzene removal catalysts, special attention should be paid to the effect of water and other impurities in the feedstock, such as SO_2_, NO_x_, Cl species, CO_2_, etc., on catalyst structure and performance. Very recently, Shen et al. reviewed research progress in VOC catalytic oxidation in simulated flue gases, emphasizing the effect of different impurities in exhaust gases on catalyst pore structure, number of active sites, and conversion efficiency [255]. The effect of water is extensively studied because it is a product of complete benzene oxidation and often present in industrial flue gases. In general, low water content (less than 1 vol.%) has no detrimental effect on VOC conversion. However, higher amounts of water vapor might adsorb on active sites and inhibit VOC adsorption; therefore, it could suppress further catalytic oxidation. Various strategies were applied for preparation of water-resistant catalysts. Wet impregnation of a Pd/Al_2_O_3_ catalyst with an Na to Pd mole ratio of 1:1 promoted water tolerance and improved low temperature benzene oxidation owing to enhanced lattice oxygen mobility [143]. A topochemical transformation route under dynamic oxygen atmosphere was used for preparation of an hierarchical Co(II)_2.8_Co(III)_1_ LDH nanostructure [256]. Presence of high valence Co ions induced excellent low-temperature reducibility and superior benzene oxidation activity (*T*_99_ = 210 °C). Addition of 3.6 vol.% water vapor slightly affected catalytic activity. Stronger adsorption of oxygen than water at a relatively high temperature was suggested owing to higher lattice oxygen mobility in Co_3_O_4_.

Modification of α-MnO_2_ via Ce and Sn deposition produced a core-shell like structure of increased Mn^3+^ content, weakened Mn-O bonds, and enhanced formation of active oxygen vacancies [257]. It was hypothesized that strong metal–support interaction (SMSI) induced ability of Sn@CeMn to activate O_2_ and H_2_O into more oxidizing (•OH) radicals, which favor water tolerance. Liu et al. prepared layered birnessite-type MnO_2_ with nanosheet morphology and modified it by Ce^3+^ and Cu^2+^ exchange [197]. A Cu–MnO_2_ catalyst exhibited the highest reaction rate and good resistance to deactivation. The best performance of this material was related to highest reducibility and lattice oxygen reactivity, leading to the highest number of active oxygen vacancies. Its considerable tolerance to water poisoning was attributed to improved surface hydrophobicity. Bimetallic Pd-Pt catalysts were deposited on mesoporous γ-Al_2_O_3_ by a high-temperature solution-phase reduction method [258]. Addition of 100 ppm chlorobenzene and 3 vol.% water during durability test at 190 °C caused a slight activity decrease due to competitive adsorption. However, the deactivation was reversible and the catalyst preserved 95% benzene conversion for 1000 h after removal of C_6_H_5_Cl and water. 

A poisoning effect of sulfur dioxide that sometimes exists in organic waste gases was also investigated. Very recently, Wang et al. reviewed the effect of SO_2_ on VOCs oxidation catalysts, addressing the main reasons for the negative impact of SO_2_ on catalyst activity, namely the competitive adsorption between SO_2_ and VOC molecules, active oxygen consumption and formation of stable metal sulfates [259]. Methods for anti-sulfur poisoning modification of diverse catalysts have been discussed and future directions for sulfur-resistant catalyst design have been proposed. Hou et al. carried out benzene oxidation over Pd_1_Co_1_/Al_2_O_3_ and Pd/Al_2_O_3_ in the presence of SO_2_ [138]. The addition of 25 ppm SO_2_ caused a sharp decrease of benzene conversion over Pd_1_Co_1_/Al_2_O_3_, but activity was gradually restored. Benzene conversion over Pd/Al_2_O_3_ gradually declined in the presence of SO_2_, and in contrast, it could not reach the initial level after SO_2_ removal. Based on XPS data, benzene temperature-programmed desorption, benzene temperature-programmed oxidation, and in situ DRIFTS, the enhanced sulfur resistance of Pd_1_Co_1_/Al_2_O_3_ catalyst was ascribed to good regeneration ability of the active sites due to rapid decomposition of sulfite or sulfate formed on the surface of single-atom dispersed palladium and cobalt. The effect of SO_2_ on benzene oxidation over CeCu mixed oxides superficially modified by Ti was studied [260]. A sample with a Ce:Cu:Ti molar ratio of 1:1:1 demonstrated optimal sulfur resistance with only 9% conversion decrease under 100 ppm SO_2_ at 360 °C within 4 h. The high sulfur resistance of this material was attributed to newly formed strong acid sites and the incremental Lewis-Brønsted acid complex at 300 °C. However, the presence of water significantly accelerated SO_2_ poisoning due to facilitated sulfate formation on reaction with hydroxy groups on the surface. Doping of Sr and Mn species positively affected sulfur resistance of 3DOM LaCoO_3_ perovskite catalysts [261]. Addition of 20 ppm of SO_2_ in the reaction mixture insignificantly changed the stability of La_0.9_Sr_0.1_Co_0.9_Mn_0.1_O_3_ catalyst with only a 2.3% decrease in benzene conversion. Modification with Sr/Mn species at the A- and/or B-site improved catalyst acidity to enhance sulfur-resisting performance. The interconnected pore structure of 3DOM increased reactant diffusion rate and reduced SO_2_ adsorption on the catalyst. Cooperation between La-Cu-Co-O perovskite and Pd in Pd/La-Cu-Co-O/cordierite catalysts prepared by a multiple-step impregnation method was found to be beneficial for stable benzene conversion in mixtures with 100 ppm SO_2_ [262]. LaCoO_3_ doping by Cu at B-sites caused decreased crystallite size of the active components. 

**Figure 13:**
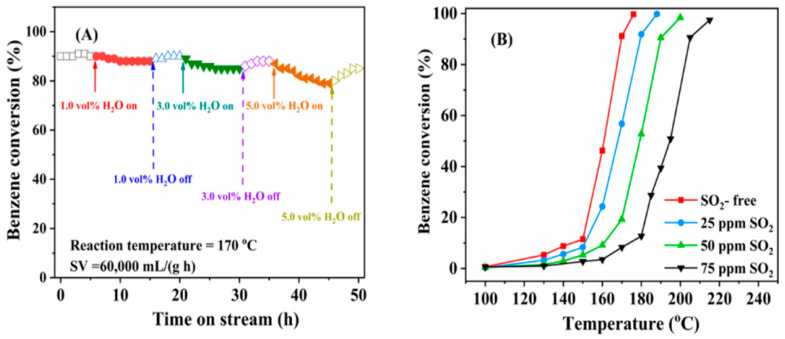
Effect of water vapor on benzene conversion over 0.94Pt-1.0rGO/α-MnO_2_ sample at 170 °C and SV = 60,000 mL·g^–1^·h^–1^ (**A**); effect of SO_2_ on benzene oxidation over 0.94Pt-1.0rGO/α-MnO_2_ sample at SV = 60,000 mL·g^–1^·h^–1^ (**B**). Reproduced from [264]. This is an open access article distributed under the Creative Commons Attribution License.

Han et al., who obtained an MnO_x_–Co_3_O_4_ interface on Ni foam, proposed a novel route for preparation of SO_2_-resistant catalysts [263]. Higher benzene conversion in the presence of 1 ppm SO_2_ was achieved over Mn_1_Co_1_-NF as compared to Co_3_O_4_-NF. Analysis of in situ DRIFT spectra and DFT calculation showed that surface metal sulfate species were preferentially formed on Mn sites rather than Co sites, thus retarding poisoning of Co–Mn interfacial active sites. Benzoquinone ring opening into maleate species on Mn_1_Co_1_–NF catalyst was slightly inhibited by addition of SO_2_. Decreased benzene conversion in the presence of SO_2_ (25, 50, or 75 ppm SO_2_) was observed over Pt supported on α-MnO_2_ nanorods promoted by reduced graphene oxide (rGO) [264]. It was suggested that generated sulfate species might cover some of the active sites, thus hindering benzene and oxygen adsorption and reaction on the catalyst surface. A sample of 0.94Pt-1.0rGO/*a*-MnO_2_ composition demonstrated a favorable effect of Pt and rGO on SO_2_ chemisorption to prevent active site blocking and enhance sulfur resistance of the catalyst (Figure 13). In summary, a decrease of specific surface area, pore size, and pore volume, and weakening of VOC adsorption capacity caused lower degradation efficiency. 

Satisfactory tolerance to chlorine poisoning was achieved through preparation of bimetallic Pt-M/Al_2_O_3_ (M = W, Mo) samples [97]. Stronger interaction between highly dispersed metallic Pt species and small MOx ensembles led to enhanced resistance of the Pt species to deactivation under Cl-containing conditions.

Coke formation on the catalyst surface and/or inside the pores is another reason for deactivation due to blocking of the active centers or hindered access of reactant molecules to adsorption sites. Some examples from the literature are noted in review papers, revealing how carbon deposition on the catalyst surface could restrict interaction between various VOCs and surface active sites, resulting in catalyst deactivation [29,41]. A limited number of works studied coke deposits formed during complete benzene oxidation. Temperature-programmed oxidation and FTIR spectroscopy were used for analysis of coke formed over Cu- and Pd-exchanged Y-type zeolites [265]. FTIR measurements revealed that polyaromatic compounds, which partly consisted of oxygenated fragments, built up insoluble coke fractions. 

**Figure 14:**
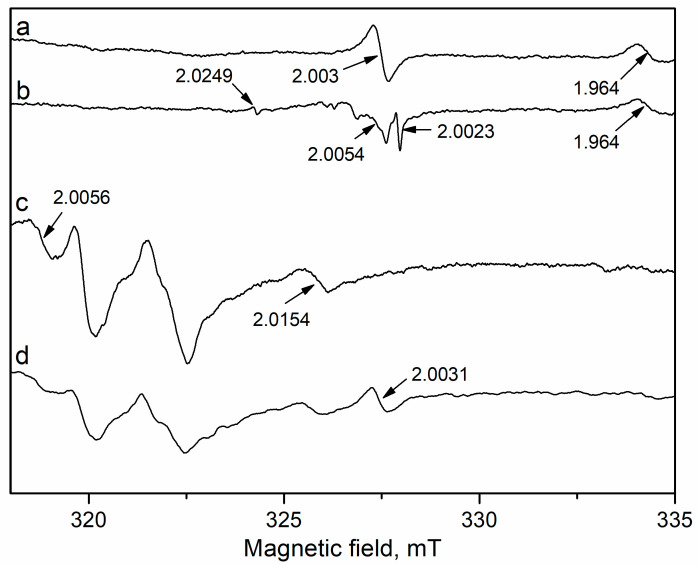
EPR spectra of samples after catalytic tests: (**a**) Au/Y-CeO_2_/Al_2_O_3_; (**b**) Pd/YCeAl; (**c**) Au/Ce, and (**d**) Pd/Ce. Reproduced from [164]. This is an open access article distributed under the Creative Commons Attribution License.

EPR spectroscopy was shown as a suitable technique to analyze formation of coke on the catalyst surface during benzene oxidation over Au and Pd catalysts on alumina-supported ceria, incl. Y_2_O_3_-doped ceria [164] in order to clarify differences in catalytic performance. The spectrum of Au/Y-CeO_2_/Al_2_O_3_ was collected because this sample demonstrated high benzene oxidation at 300 °C within 24 h (Figure 14, line a). The products were only CO_2_ and water, but the conversion degree was 90%. A single EPR line with g factor 2.003, which is characteristic of carbon-centered radicals and coke, was recorded. The same signal was detected in the spectrum of Pd/CeO_2_ (Figure 14, line d) that showed lower activity in comparison with Au/CeO_2_ (Figure 14, line c). Coke formation could be the reason for the observed catalyst behavior. In the EPR spectrum of the best-performing Pd/Y-CeO_2_/Al_2_O_3_ sample, another line was registered related to carbon-centered radicals with g-value of 2.0023, typical of free electrons (Figure 14, line b). Some products of mild oxidation after 24 h in stream were detected in the spectrum of this sample. According to Green et al., g-values close to that of free electrons were attributed to organic radicals [266]. An EPR line of g value of 2.0054 was registered in the spectrum of Pd/Y-CeO_2_/Al_2_O_3_, indicating the presence of oxygen-centered radicals.

## 7. Conclusions and Future Directions

This review summarizes progress over the past decade made in developing highly efficient catalysts for effective elimination of benzene at low temperatures. Being VOCs, benzene, toluene, ethylbenzene, and xylene belong to a group of compounds known as BTEX that are regarded as the most common aromatic VOCs with significant contribution to industrial emissions. Benzene is classified as one of the most hazardous air pollutants among non-halogenated aromatic hydrocarbons with toxic, carcinogenic, and mutagenic effects. Complete oxidation to CO_2_ and water is an attractive approach to benzene removal due to high efficiency, low energy consumption, and absence of secondary pollution. Many studies select benzene as a target molecule for testing catalyst activity in aromatic hydrocarbon oxidation because the extraordinary stability of the six-membered ring structure represents a great challenge.

Two main types of catalysts, specifically supported noble metals and non-noble metal oxides or mixed metal oxides, have been most intensively studied for application in complete benzene oxidation. Generally, the performance of supported noble metal (e.g., Pt, Pd, Rh, Ru, Au, and Ag) catalysts is governed by particle dispersion, chemical state, and location of active sites, which are influenced by various factors such as preparation method, precursor type, particle morphology, and nature of the carrier. Selection of supporting material with suitable physicochemical properties, such as surface area, acid/basic characteristics, reducibility, and ability to enhance surface oxygen mobility is an important task since it can strongly affect nanoparticle size, loading, and dispersion as well as potentially controlling catalyst activity and stability. The relevance of using various oxides, namely Al_2_O_3_, CeO_2_, TiO_2_, Fe_2_O_3_, either pure or modified by other metal ions, was demonstrated. The benefit of using perovskites or functionalized ZSM-5 and SBA-15 was also revealed. A promising application of diatomite, hydroxyapatite waste eggshells, soybean straw, coal fly ash zeolites, and pearl shell powder from the pearl industry as cheaper, environmentally friendly, and cost-effective carriers was based on the strategy of green and sustainable chemistry. Optimization of noble metals utilization through alloying was discussed as an approach to overcome concerns with high cost and limited availability of noble metals. Preparation of bimetallic catalysts was another way to boost benzene oxidation and overcome the problem with precious metal nanoparticle aggregation at higher temperatures. 

The most commonly used non-noble metal-based catalysts include transition and rare earth metal oxides, owing to their ability to form oxygen vacancies. Among them, Mn, Co, Cu, and Ce oxide-based catalysts were mostly reported in the literature for benzene oxidation. Manganese oxides (MnOx) demonstrated a great potential for catalytic degradation of VOCs due to numerous advantages, such as multivalent oxidation states, different crystal phases and morphologies, good redox and catalytic properties, long-term durability, low cost, and abundance in nature. The advantageous features of Co_3_O_4_ were high reducibility, abundance of oxygen vacancies able to provide active surface oxygen species, and unique textural properties, as well as low cost and chemical and thermodynamic stability. A promising performance for benzene removal was exhibited by compositions of mixed CuO with other metal oxides because pure CuO is prone to deactivation owing to its low thermal stability. The advantage of employing CeO_2_ as a component and promoter for oxidation reactions was related to Ce^4+^ ↔ Ce^3+^ fast transfer assuring oxygen storage and supply. Many works highlighted the role of surface layers of oxygen and ceria vacancies for catalytic activity by addressing the importance of synergistic effects between components, and oxygen-vacancy defect engineering. Mixed metal oxides of more complex composition were also adopted for complete benzene oxidation. In all cases, emphasis was given to designing catalysts of high specific surface area, improved low temperature reducibility and oxygen mobility, and availability of weakly bonded oxygen species.

An important question concerning industrial activities is related to the presence of more than one pollutant in waste emissions. Some examples of benzene oxidation in mixtures over different catalytic materials were discussed, considering different mutual effects such as suppression of benzene oxidation or promotional effects of benzene on other VOC oxidation processes.

Three different reaction mechanisms, namely Langmuir–Hinshelwood, Eley–Rideal, and Mars–van Krevelen were considered in the case of benzene oxidation depending on catalyst composition, and reaction conditions. Characterization techniques such as in situ FTIR/DRIFTS, EPR, and Raman spectroscopy were employed to detect active oxygen species, migration, and transformation of intermediate species as well as final products for benzene oxidation over the catalytic materials. Theoretical modeling and DFT calculations also provided useful information about catalyst design and prediction of catalyst performance. 

A challenge to preparation of highly efficient benzene removal catalysts was resistance ability in the presence of water and other impurities in the feedstock, such as SO_2_, Cl species, CO_2_, etc., in exhaust gases as well as coke formation. Many efforts have been made to disclose the mechanisms underlying catalyst deactivation and, in this connection, progress in designing materials of high stability was reexamined. 

This review revealed that although a great deal of work has been achieved to date in rational design of benzene oxidation catalysts, there remain yet some challenges. The combination of material composition optimization with suitable morphological control and correlation between physicochemical properties and catalytic performance will continue to guide research efforts in the development of new complete benzene oxidation catalysts. 

Future research could be focused on the following issues.

Among the variety of factors affecting catalyst performance, special attention is paid to the role of oxygen vacancies. Very recently, a comprehensive review summarized recent progress in oxygen vacancy engineering for VOC oxidation, addressing strategies of oxygen vacancy formation, characterization methods and impact of oxygen vacancies on VOC catalytic removal [267]. Future research efforts should enable controllable oxygen defect formation and clarify the effect of oxygen vacancy location on the complete benzene decomposition mechanism.

New investigations should consider elaboration of SACs potential for effective benzene elimination owing to unequaled advantages of these catalysts such as full exposure of active sites, tunable coordination, and strong metal-support interaction. Despite favorable features, only a few papers studied catalytic performance of SACs for benzene removal [116,138,170,268]. Synthesis strategies and catalyst composition face considerable challenges. Benzene’s adsorption and reaction behavior are complicated because it is a large molecule. A combination of theoretical calculations and sophisticated in situ techniques will provide insight for understanding of the structure–activity relationship of SACs. 

Implementation of catalytic oxidation in the presence of an electric field could be investigated owing to low energy consumption and high activity for benzene removal. The advantage of microwave-assisted reactions for effective degradation of benzene has been very recently reported [195,269]. Improved benzene oxidation efficiency provided experimental confirmation that coupling catalytic oxidation and the microwave approach is a promising technology for elimination of low concentrations of benzene emitted from small- and medium-scale sources.

In many cases, high space velocities during benzene oxidation over granulated catalysts caused a very high pressure drop or active phase loss. From a practical point of view, considering favorable features of structured catalysts such as monoliths, foams, or microreactors for benzene removal, more attention should be focused on further investigations in this field. 

## Figures and Tables

**Table 1:** Platinum-based catalysts for complete benzene oxidation.

Catalyst	Preparation Method	Benzene Concentration (ppm)	Space Velocity	Oxidation Efficiency ^1^ T_90_/°C	Ref.
0.63 Pt/Al_2_O_3_	Reduction of Pt(acac)_2_ with oleylamine, adsorption	100	60,000 mL·g^–1^·h^–1^	108	[93]
1 Pt/Al_2_O_3_	Reduction with NaBH_4_	1000	50,000 h^–1^	186	[94]
1 Pt/Al_2_O	Reduction with H_2_	1000	50,000 h^–1^	246	[94]
1 Pt/Al_2_O	Impregnation	1000	50,000 h^–1^	269	[94]
1 Pt/Al_2_O_3_	Modified EG reduction	2800	32,000 mL·g^–1^·h^–1^	144	[95]
0.3 Pt/10Ce-10V/Al_2_O_3_	Impregnation	1000	20,000 h^–1^	235	[96]
PtW/Al_2_O_3_	Solvothermal synthesis	1000	40,000 mL·g^–1^·h^–1^	140	[97]
1 Pt-0.6rGO/Al_2_O_3_	Reduction with NaBH_4_	100	60,000 mL·g^–1^·h^–1^	135	[98]
1 Pt/meso CeO_2_	Reduction with NaBH_4_	2 g·m^−3^	48,000 mL·g^–1^·h^–1^	153	[99]
1 Pt/CeO_2_	Reduction with H_2_	40	6000 mL·h^–1^	130	[100]
0.2 Pt_NP_/Fe_2_O_3_	Wet impregnation with NaBH_4_ reduction	0.05 vol% C_6_H_6_, 20 vol% O_2_, N_2_	60,000 mL·g^–1^·h^–1^	165	[101]
0.2 Pt_single atom_/Fe_2_O_3_	Wet impregnation with NaBH_4_ reduction	0.05 vol% C_6_H_6_, 20 vol% O_2_, N_2_	60,000 mL·g^–1^·h^–1^	225	[101]
0.2 Pt_nanocluster_/Fe_2_O_3_	Wet impregnation with NaBH_4_ reduction	0.05 vol% C_6_H_6_, 20 vol% O_2_, N_2_	60,000 mL·g^–1^·h^–1^	335	[101]
0.25 Pt/3D meso Fe_2_O_3_	Reduction during synthesis with NaBH_4_	1000	20,000 mL·g^–1^ h^–1^	198	[102]
1 Pt/TiO_2_-reduced	Incipient wetness impregnation	100	8696 h^–1^	167	[103]
0.3 Pt/diatomite	Bioreduction	1000	60,000 mL·g^–1^·h^–1^	195	[105]
0.8 Pt/eggshell	Bioreduction	1000	80,000 mL·g^–1^·h^–1^	178	[106]
0.5 Pt/soybean straw	Reduction during synthesis with NaBH_4_	1000	120,000 mL·g^–1^·h^–1^	179	[107]
0.5 Pt/ fly ash zeolite X	Impregnation	4200 mg·m^−3^	4000 h^–1^	~235	[108]
1 Pt-2Co/Sb doped SnO_2_	Impregnation Reduction with H_2_	500	60,000 mL·g^–1^·h^–1^	165	[109]
0.5 Pt-Fe/Al_2_O_3_	Wet impregnation	1000	60,000 mL·g^–1^·h^–1^	~170	[110]
0.66 Pt/TS-1	Incipient wetness impregnation	120	60,000 mL·g^–1^·h^–1^	130	[111]
0.5 Pt/ZSM-5	Reduction during synthesis with NaBH_4_	1000	20,000 mL·g^–1^·h^–1^	178	[112]
0.5 Pt/ZSM-5	Incipient wetness impregnation	4200 mg·m^−3^	4000 h^–1^	189	[113]
Pt/SBA-15	Impregnation and reduction with NaBH_4_	1000	60,000 mL·g^–1^·h^–1^	145	[114]
2 Pt@Ti_3_C_2_	Impregnation	10	3000 mL h^–1^	162	[115]
0.0383 Pt/OMS-2	PVA protected reduction with NaBH_4_	1000	20,000 mL·g^–1^·h^–1^	189	[116]
0.56 Pt/meso-CoO	polyvinyl alcohol-assisted reduction	1000	80,000 mL·g^–1^·h^–1^	186	[117]
0.2 Pt/MCM-41		1000	20,000 mL·g^–1^·h^–1^	207	[119]

^1^ Temperature at which benzene conversion reaches 90%.

**Table 2:** Palladium-based catalysts for complete benzene oxidation.

Catalyst	Preparation Method	Benzene Concentration (ppm)	Space Velocity	Oxidation Efficiency ^1^ T_90_/°C	Ref.
0.2 Pd/6Ce-pearl shell powder	Impregnation, reduction with N_2_H_4_.xH_2_O	1000	20,000 h^–1^	~285	[120]
0.2 Pd/6La/ZSM-5	Treatment with IR lamp, reduction with N_2_H_4_.xH_2_O	1000	20,000 h^–1^	~250	[121]
0.16 Pd-5.12Ni/SBA-15	Impregnation, HCl treatment at RT	1000	120,000 mL·g ^–1^·h^–1^	~245	[122]
0.96 Pd/Mn_3_O_4_-	Impregnation-bioreduction	1000	120,000 mL·g ^–1^·h^–1^	207	[123]
0.5 Pd/shrimp waste-600	Sol-immobilization	1000	60,000 mL·g ^–1^·h^–1^	220	[124]
0.93 Pd/mesoCoO	In situ reduction H_2_	1000	40,000 mL·g^–1^·h^–1^	189	[127]
0.3 Pd/SBA-15	Impregnation, reduction H_2_	880	26,000 h^–1^	~240	[129]
PdOx−WOx−TiO_2_	Impregnation	1000	40,000 mL·g^–1^·h^–1^	200	[130]
1 Pd-5Mo/Al_2_O_3_	Incipient wetness impregnation	0.2 vol*%* C_6_H_6_, 20 vol*%* O_2_, N_2_	4800 h^–1^	190	[133]
0.3 Pd-10Ce/Silica- pillared clays	Impregnation	1000	20,000 h^–1^	*T*_100_*/*280	[135]
0.43 Pd-0.13Co/Al_2_O_3_	PdCo NPs mixed with γ-Al_2_O_3_	1000	40,000 mL·g^–1^·h^–1^	250	[138]
0.5 Pd/kit-CeO_2_	Biogenic synthesis	1000	20,000 mL·g^–1^·h^–1^	187	[139]
1 Pd/waste red mud	Incipient wetness impregnation	1000	75,000 h^–1^	~250	[140]
0.5 Pd-CeMnO_3_	NaBH_4_ reduction impregnation	500	20,000 mL·g^–1^·h^–1^	186	[141]
1 Pd/Ce_0.25_Co_0.75_	Self-propagating combustion	0.1 vol% C_6_H_6_, 10 vol% O_2_, N_2_	60,000 h^–1^	185	[142]
1 Pd-Na/Al_2_O_3_	Wet impregnation	1500	90,000 mL·g^–1^·h^–1^	193	[143]
0.2 Pd/AlNi-pillared clays	One-step high-temperature solution-phase reduction	1000	20,000 h^–1^	240	[144]

^1^ Temperature at which benzene conversion reaches 90%.

**Table 3:** Gold-based catalysts for complete benzene oxidation.

Catalyst	Preparation Method	Benzene Concentration (ppm)	Space Velocity	Oxidation Efficiency T_90_/°C	Ref.
3 Au-4V_2_O_5_/CeO_2_	Deposition-precipitation	4200 mg·m^−3^	4000 h^–1^	175	[147]
3 Au-4MoO_3_/CeO_2_	Deposition-precipitation	4200 mg·m^−3^	4000 h^–1^	160	[148]
Au/BSA-CeO_2_	Deposition-precipitation	1000	20,000 mL·g^–1^·h^–1^	210	[149]
Au/Nb-CeO_2_	Deposition-precipitation	1000	30,000 mL·g^–1^·h^–1^	258	[150]
3 Au/CeO_2_–10CoO_x_	Deposition-precipitation	4200 mg·m^−3^	4000 h^–1^	150	[151]
6.5 Au/meso-Co_3_O_4_	Impregnation	1000	20,000 mL·g ^–1^·h^–1^	189	[155]
Au/*h*-Fe_0.18_Co_2.82_O_4_	Deposition-precipitation	1200	4000 h^–1^	184	[156]
5 Au/*β*-MnO_2_	Deposition-precipitation with NaOH	2000	60,000 mL·g^–1^·h^–1^	~225	[157]
Au/SnO_2_ plates	Deposition-precipitation	2000	3600 mL·h^–1^	~375	[159]
2 Au/Cu-Ce/Al_2_O_3_	Deposition-precipitation	4200 mg·m^−3^	4000 h^–1^	235	[160]
1 Au/CeO_2_-HAP	Deposition-precipitation	120	30,000 h^–1^	~220	[161]

**Table 4:** Silver-based catalysts for complete benzene oxidation.

Catalyst	Preparation Method	Benzene Concentration (ppm)	Space Velocity	Oxidation Efficiency ^1^ T_90_/°C	Ref.
2 Ag/Co_3_O_4_	Solvothermal method	100	120,000 mL·g ^–1^·h^–1^	201	[165]
9 Ag/Co_3_O_4_ nanofiber		200	60,000 mL·g ^–1^·h^–1^	183	[166]
Ag/CeO_2_-Co_3_O_4_	One-pot solvothermal method	100	66,000 mL·g ^–1^·h^–1^	193	[167]
Ag-MnO_x_	Reduction	200	60,000 mL·g^–1^·h^–1^	203	[168]
K/Ag-OMS-40	Hydrothermal method	1500	90,000 mL·g^–1^·h^–1^	~200	[169]
Ag/HMO	Hydrothermal	200	23,000 h^−1^	~200	[170]
Ag-Mn/ZrO_2_	Adsorption	395	12,000 mL·g^–1^·h^–1^	~350	[171]
AgNi/BCN-400	Impregnation	1400 mg·m^−3^	not reported	25	[172]
Ag/ZSM-5/SBA-15	Reduction NaBH_4_	50 mL*·*min^−1^ O_2_/C_6_H_6_	30,000 mL·g^–1^·h^–1^	~275	[173]
0.025 Ag-H_3_PW_12_O_40_- LiCoO_2_	Thoroughly ground and thermally treated	450–480	120,000 mL·g ^–1^·h^–1^	275	[174]
19.9 Ag/eggshell	Impregnation	1000	20,000 mL·g ^–1^·h^–1^	225	[175]

^1^ Temperature at which benzene conversion reaches 90%.

**Table 5:** Non noble metal-based catalysts for complete benzene oxidation.

Catalyst	Preparation Method	Benzene Concentration (ppm)	Space Velocity	Oxidation Efficiency ^1^ T_90_/°C	Ref.
3D Mn_2_O_3_ cube-like	Hydrothermal method	500	6000 mL·h^–1^	248	[181]
Mn_2_O_3_	Citric acid solution combustion	200	60,000 mL·g^–1^·h^–1^	212	[182]
λ-MnO_2_	Acid etching of ZnMn_2_O_4_	500	60,000 mL·g ^–1^·h^–1^	170	[183]
Birnessite-type MnO_2_	Redox reaction between KMnO_4_ and CH_3_OH	395	120,000 mL·g^–1^·h^–1^	175	[185]
MnTiO_x_	Hydrothermal method	1000	45,000 mL·g^–1^·h^–1^	258	[186]
10 MnO*_x_*/TiO_2_	Impregnation	500	60,000 mL·g^–1^·h^–1^	~300	[187]
Mesoporous Cu_0.6_Mn	Co-nanocasting using SBA-15	500	3150 mL·g^–1^·h^–1^	234	[189]
Cu/Mn-2	Precipitation	1000	~21,740 mL·g^–1^·h^–1^	219	[192]
CuMnO_2_	Citric acid sol-gel	100	60,000 mL·g^–1^·h^–1^	186	[194]
Cu-Mn spinel oxides	Coprecipitation	400	60,000 mL·g^–1^·h^–1^	~125	[195]
Mn_5_Co_5_ nanorod	Sol–gel chelating	1000	120,000 mL·g^–1^·h^–1^	237	[198]
1D α-MnO_2_@Co_3_O_4_	Hydrothermal method	1000	120,000 mL·g^–1^·h^–1^	247	[199]
6Mn4Ce/silica spheres	Oxalate route	100	6000 mL·h^–1^	216	[202]
10 MnCeOx (9:1)/NaY-type zeolite	Impregnation	1000	20,000 h^–1^	~240	[203]
K-doped Mn_5_Ce_5_	Precipitation	1000	20,000 mL·g^–1^·h^–1^	202	[204]
0.2 Ce/MnAl LDH	Co-precipitation	100	60,000 mL·g^–1^·h^–1^	210	[207]
Mn_4_Ni_1_	Oxalate method	200	60,000 mL·g^–1^·h^–1^	172	[209]
Sm_0.01_-todorokite MnO_2_	Hydrothermal	237	120,000 mL·g^–1^·h^–1^	*T*_100_*/*175	[211]
Sn/α-MnO_2_	Redox with Sn^4+^ addition	450	120,000 mL·g^–1^·h^–1^	200	[212]
Sr-OMS	Redox reaction	2 g m^−3^	48,000 mL·g^–1^·h^–1^	211	[213]
Co-Al hydrotalcite-derived	Precipitation	516	36,000 mL·g^–1^·h^–1^	257	[218]
70Co-30Ce	Mechanochemical mixing	42 g m^−3^	4000 h^–1^	175	[220]
Cu_0.5_Co_2.5_Al	Co-precipitation	1000	60,000 mL g^–1^ h^–1^	290	[223]
10 CuO-CeO_2_ nanofibers	Electrospinning	500	3000 mL h^–1^	437	[226]
Ce-MOF	Thermal decomposition	1000	20,000 mL g^–1^ h^–1^	240	[228]
12.5 CeMnO_x_	Flame spray pyrolysis	1000	60,000 mL g^–1^ h^–1^	250	[230]
10 Ce/ITQ-2 zeolite	Impregnation	1.3	12,000 mL g^–1^ h^–1^	350	[234]

^1^ Temperature at which benzene conversion reaches 90%.

## Data Availability

No new data were created in this study. The data presented in this study are available in journals indexed by Scopus and Web of Science.
